# Pennsieve: A Collaborative Platform for Translational Neuroscience and Beyond

**DOI:** 10.1038/s41597-025-06075-5

**Published:** 2025-11-19

**Authors:** Zack Goldblum, Zhongchuan Xu, Haoer Shi, Patryk Orzechowski, Jamaal Spence, Kathryn A. Davis, Brian Litt, Nishant Sinha, Joost Wagenaar

**Affiliations:** 1 Center for Neuroengineering and Therapeutics, University of Pennsylvania, Philadelphia, US; 2 Department of Bioengineering, School of Engineering and Applied Sciences, University of Pennsylvania, Philadelphia, US; 3 Department of Biostatistics, Epidemiology and Informatics, University of Pennsylvania, Philadelphia, US; 4 Department of Automatics and Robotics, AGH University of Krakow, Kraków, Poland; 5 Department of Neuroscience, Perelman School of Medicine, University of Pennsylvania, Philadelphia, US; 6 Department of Neurology, Perelman School of Medicine, University of Pennsylvania, Philadelphia, US

**Keywords:** Data publication and archiving, Hardware and infrastructure, Neuroscience, Computational platforms and environments, Data integration

## Abstract

The exponential growth of neuroscientific data necessitates platforms for data management and multidisciplinary collaboration. In this paper, we introduce Pennsieve, an open-source, cloud-based scientific data management platform that supports findable, accessible, interoperable, and reusable (FAIR) data sharing. It has integrated tools for data visualization, processing, and peer-reviewed data publishing that promote collaborative research and high-quality datasets optimized for downstream analysis, both in the cloud and on-premises. Pennsieve welcomes data submissions from individual investigators and small labs through entire consortia. It already serves more than 80 research groups worldwide and forms the core for several large-scale, interinstitutional projects and major government neuroscience research programs. Pennsieve stores over 125 TB of scientific data, with 35 TB of data publicly available in more than 350 high-impact datasets. By facilitating scientific data management, discovery, and analysis, Pennsieve fosters a robust and collaborative research ecosystem for neuroscience and beyond.

## Introduction

Scientific data management systems are tasked with accommodating the evolving data integration needs of researchers, where detailed metadata and annotations play a crucial role in interpreting complex datasets^1–3^. This is especially challenging in the neurosciences where the majority of datasets are increasingly multimodal, often spanning multiple domains including neuroimaging, electrophysiology, the electronic medical record, as well as immune and genetic information^4,5^. The current neuroscience data landscape is highly fragmented and generally organized into propriety formats and modality-specific archives that do not communicate with each other^6^. While this provides in-depth capabilities for specific domains, it can inadvertently create data silos that hinder large-scale research efforts^7–9^. As a consequence, these data repositories are underutilized and their potential for scientific discovery is greatly diminished. Improving the research potential of these repositories will require new approaches to data curation that can ingest and link disparate data to facilitate cross-modal analysis and discovery^10^.

As a field, neuroscience is undergoing a significant transition from primarily closed to open science^6,11^. This shift, driven in large part by data sharing policies from NIH and the EU^12^, is part of a broader transformation involving advances in research computing infrastructure and neuroscience-specific data standards^13–15^. The resulting exponential increase in neuroscience data volume and complexity presented researchers with unprecedented challenges in data management and cross-institutional collaboration^16–18^. As datasets expanded in scale from gigabytes to terabytes and even petabytes^19^, traditional methods of data storage, sharing, and analysis became inadequate, particularly for collaborative efforts spanning multiple institutions. Such challenges underscored the need for a standardized approach to data sharing, leading to the widespread adoption of the FAIR (Findable, Accessible, Interoperable, and Reusable) principles^20^. The FAIR principles have become a major framework for data interoperability and reproducible research^21^.

To further improve the research potential of scientific data, it needs to be standardized, curated with detailed metadata and annotations, and made accessible to both researchers and automated methods^22,23^. These needs call for platforms that can accommodate the scale and complexity of neuroscience data while supporting the collaborative research necessary for discovery and innovation in neuroscience^24–26^.

In this paper, we introduce Pennsieve, a scalable, cloud-based platform for modern scientific data management. Pennsieve’s design prioritizes data curation, metadata management, collaborative research, and data publication. Adhering to the FAIR principles of data sharing, Pennsieve ensures that datasets are not only well-managed but also broadly accessible for future research in neuroscience. Below, we will explore current opportunities in neuroscience data management, highlight the landscape of data repositories, and demonstrate how the design of Pennsieve enables a collaborative research ecosystem.

## Results

### Current Opportunities for Neuroscience Data Management

Properly managing neuroscience data must addresses the challenges presented by large-scale, diverse datasets. Neuroscience research is becoming increasingly data-driven, a trend accelerated by advances in acquisition techniques and computational capabilities. This provides new opportunities to manage these data in ways that catalyze scientific discovery:

1. **Multimodal Data Management**: Multimodal data enables richer analyses of brain function and behavior by allowing researchers to investigate relationships between different data types^1,4,27^. Effective neuroscience data management systems should facilitate integrating, storing, and analyzing diverse data modalities.
2. **Comprehensive Metadata Support**: Integrating metadata and annotations with data is important for understanding and reproducing neuroscience research^28,29^. This makes datasets accurately described, easily searchable, and properly contextualized for reuse by other researchers.
3. **FAIR Data Sharing**: The FAIR principles ensure that data is well-managed and can be easily shared and reused by others^20^. Adopting these principles enables researchers to integrate data from various sources and leverage it for greater discovery^30^.
4. **Optimizing Data Reliability and Utilization**: Ensuring data quality and making it readily accessible to processing and analysis tools supports reproducibility efforts^3,31^. Datasets should be structured and easy to download and use, both through the web and programmatically.
5. **Facilitating Data Integration and Standardization**: The generation of disparate neuroscience datasets complicates data integration and standardization^25,28^. When data repositories adopt standardization practices, it improves data interoperability and reuse^21,32,33^.
6. **Fostering Collaborative Science**: Isolated data management systems limit collaborative research. Integrated platforms that facilitate data sharing and cross-disciplinary efforts help build a research ecosystem^34^.
7. **Enabling Scalable Analysis**: With scale, data analysis solutions need to become more integrated with data management platforms^35,36^. No longer is it sufficient to just support data sharing with the intent to enable downloading files.
8. **Ensuring Resource Sustainability**: Building a proof of concept is very different from building a lasting resource that people want to use. Sustainable platforms must handle long-term data storage, scale with increasing user engagement, and implement revenue models that recoup operational costs^37,38^.

These opportunities are interrelated and should be considered collectively as ways that platforms can contribute to a more robust and efficient research ecosystem. The implementation of FAIR principles underpins efforts to enhance data usability, reliability, and standardization, which in turn facilitates integrating and reusing multimodal datasets. Concurrently, sustainable data resources that support collaboration and scalable analyses enable large-scale research efforts.

### Neuroscience Platforms Overview

Several platforms have been developed to meet the needs of neuroscience researchers, each offering unique features and capabilities tailored to different aspects of data management and analysis. Common across these platforms is a commitment to the FAIR principles and data standardization, though implemented to varying degrees. Challenges remain in comprehensive metadata curation, seamless data integration across modalities, and collaborative research environments. As the volume and complexity of neuroscience data continue to grow, issues of scalability and usability become increasingly important.

The following survey of prominent neuroscience data repositories showcases a diverse research ecosystem that reflects the breadth of requirements in neuroscience research. Understanding this landscape contextualizes Pennsieve’s unique approach to curating data, managing metadata, and accelerating collaborative research.

#### Brain-CODE

Brain-CODE^39^ is a neuroinformatics platform developed by the Ontario Brain Institute (OBI) to manage, analyze, and share multi-dimensional data across different brain conditions. Its federated approach to integrating data allows it to link with other platforms and national databases such as REDCap. It primarily collects neuroimaging, clinical, and multiomics data with Common Data Elements (CDEs) provided by Brain-CODE standardizing data across research projects. The platform leverages a centralized, high-performance computing environment that offers analytical tools and scalable resources with virtual workspaces for data analysis.

#### brainlife.io

brainlife.io^36^ is dedicated to reproducible neuroscientific analysis. It combines high-performance computing and cloud resources to run specialized applications for various neuroimaging processing and analysis tasks. These applications can be combined into workflows and used in collaborative research efforts with shared computational resources. Built-in features support reproducible analysis and a standardized ‘Datatype’ format allows interoperability between applications. With its public-funding model, brainlife.io can work jointly with other public platforms and support their resources.

#### The Data Archive for the BRAIN Initiative (DABI)

DABI^40^ serves as a specialized repository for human invasive neurophysiology data derived from NIH Brain Research Through Advancing Innovative Neurotechnologies (BRAIN) Initiative projects^41^. DABI allows users to upload different data types, including electrophysiology, imaging, pathology, demographic, behavioral data, and surgical notes. While DABI recommends Brain Imaging Data Structure (BIDS)^42^ and Neurodata Without Borders (NWB)^43^ formats, it does not strictly require them. The platform offers both centralized and cloud-based storage options, implements data de-identification processes, and provides integrated analytical tools for intracranial EEG (iEEG) data analysis.

#### Distributed Archives for Neurophysiology Data Integration (DANDI)

DANDI^44^, also supported by the BRAIN Initiative, is a cloud-based public archive and collaboration platform designed for cellular neurophysiology data sharing, archival, and analysis. DANDI uses the NWB standard as its core data format for interoperability across different neurophysiology data types. The platform offers a JupyterHub web interface for exploring data, and supports data streaming and compute-near-data functionality to work with large datasets in the archive. This programmatic access to the data and metadata encourages secondary research use.

#### EBRAINS

EBRAINS^45^, developed through the EU-funded Human Brain Project (HBP)^46^, is a research infrastructure designed to integrate diverse neuroscience data, tools, and models into a cohesive platform. It gathers data from different modalities and species, hosting not only common data types like MRI and EEG, but also brain atlases, models, and software. There is a significant emphasis on microscopy scans and atlases as well. EBRAINS is compliant with the FAIR principles and has adopted the open Metadata Initiative for Neuroscience Data Structures (openMINDs) framework for metadata management^47^. The platform aims to improve collaborative brain research by providing tools and resources, including access to high-performance compute.

#### The Image and Data Archive (IDA)

The IDA^48^ is a platform designed for exploring, archiving, and sharing neuroscience data. Managed by the Laboratory of Neuro Imaging (LONI) at the University of Southern California, the IDA has a global reach, facilitating large-scale, multi-site collaborations. The IDA supports a range of data types, including neuroimaging, biospecimen, and genetic data. It provides integrated tools for data de-identification and search capabilities to create custom data collections. With ongoing support from NIH, among other organizations, the IDA remains an impactful resource for preserving and sharing research data.

#### OpenNeuro

Finally, OpenNeuro^49^ is a platform that promotes free and open sharing of neuroscience data. Its minimal restrictions on data access and adherence to the FAIR principles enables data reuse by researchers. Initially focused on fMRI data, it has expanded to include other modalities such as EEG, iEEG, MEG, and PET. OpenNeuro only accepts data in the BIDS format to maximize compatibility and uniformity across its database. While the platform itself focuses on managing and sharing data rather than analysis, it partners with other platforms (including brainlife.io) that provide cloud-based tools for data analysis and visualization.

### Pennsieve Platform

**Fig. 1:**
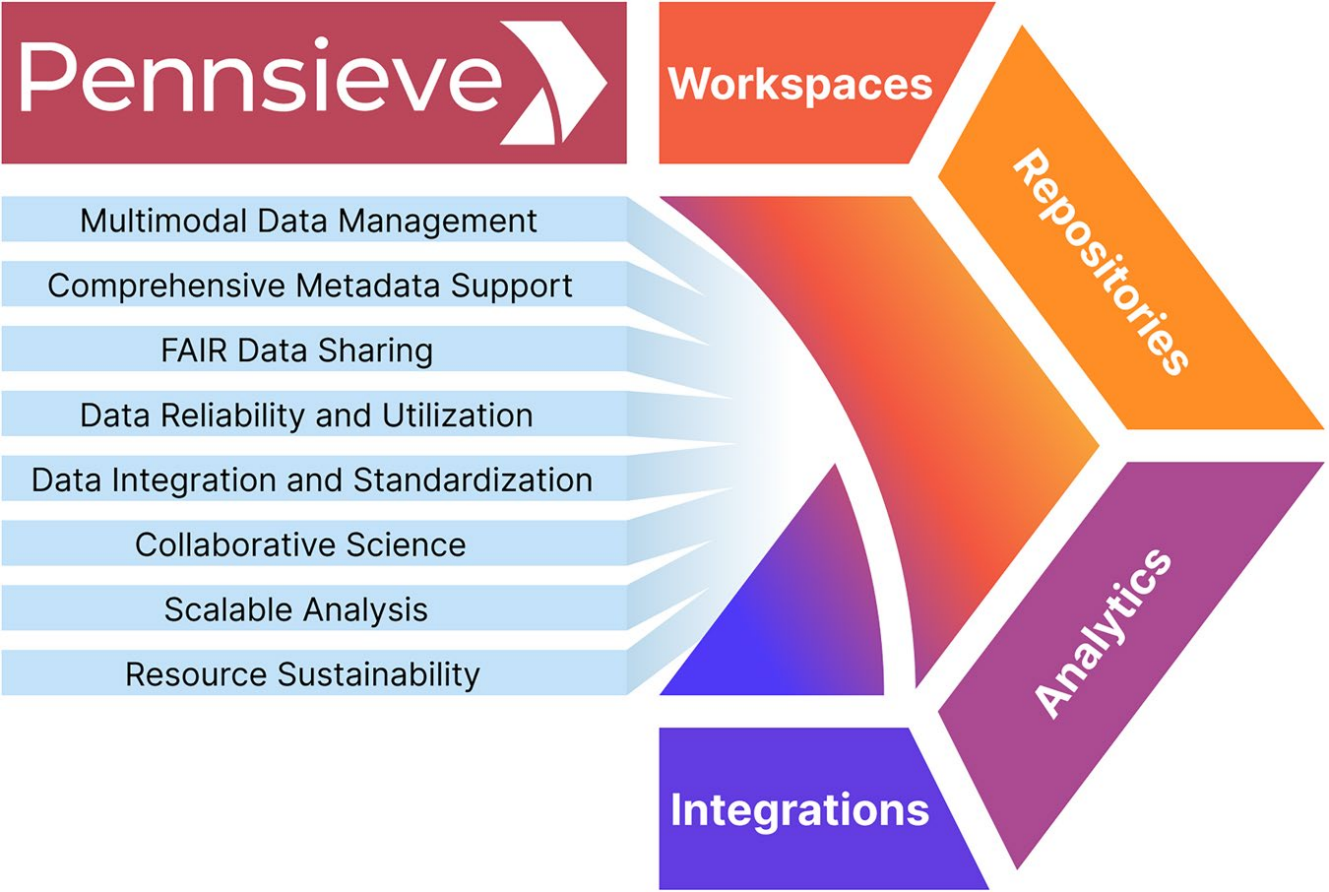
The Pennsieve platform serves as a data management infrastructure for the global scientific community. It is built around shared workspaces, data repositories, scalable analytics, and integrations that facilitate collaborative research at scale. *FAIR: Findable, Accessible, Interoperable, and Reusable*.

Pennsieve is an open-source, cloud-based scientific data management platform (Fig. 1). It was originally developed for industry under the name Blackfynn Inc. as a cloud-based platform for scientific data management that could support multiple large-scale collaborations. Based on its academic predecessor (IEEG.org)^17^, Blackfynn was funded by DARPA, NIH, and other sources to develop a sustainable, scalable platform for data management and integration for the neurosciences. In 2021, the platform transitioned to an open-source model under the name Pennsieve. It is currently developed and supported as an academic data sharing and integration platform at the University of Pennsylvania.

Pennsieve is a mature platform built on cloud resources that ensure scalability, availability, and security. The set of features that distinguish Pennsieve are multimodal data management, flexible metadata schemas, data curation and governance protocols, peer-reviewed data publishing mechanisms, and a scalable, sustainable architecture suited for high-impact scientific research.

At its core, Pennsieve differentiates between two types of datasets: *private* datasets that are shared within a workspace on the platform, and *published* datasets that are publicly available, versioned, and associated with a Digital Object Identifier (DOI) for citation in publications. Users can belong to multiple workspaces and create datasets that are selectively shared with other workspace members. Several investigator groups leverage this functionality for collaborative efforts while maintaining data privacy. For fully curated datasets ready for publication, Pennsieve has mechanisms that generate versioned snapshots of the dataset that can be made publicly accessible through Pennsieve Discover, the platform’s dedicated public data repository. This functionality is used by multiple NIH programs to support FAIR sharing of scientific data.

In the following sections, we demonstrate how Pennsieve aligns with the previously identified opportunities for neuroscience data management.

#### Multimodal Data Management

Central to Pennsieve’s design is the philosophy that integrating file management with metadata management is crucial for capturing scientific data in its full context. This guiding principle is reflected in the platform’s support for diverse data modalities and project structures. These encompass clinical, imaging, timeseries, and molecular data, along with associated metadata (Fig. S6). To make full use of this support, users are provided with a suite of features for dataset control, including creation, deletion, file organization, sharing, and permissions management (Fig. S4). For programmatic data management, Pennsieve’s API enables operations spanning from data upload and download to complex query and retrieval tasks. Furthermore, datasets can be categorized into collections and assigned status indicators that reflect their progress within publishing pipelines.

#### Comprehensive Metadata Support

Pennsieve provides functionality for comprehensive metadata management with an array of tools designed to capture, organize, and visualize them effectively. A Pennsieve dataset consists of both a folder/file structure and a metadata graph which can reference each other. Users can create detailed metadata models with custom schemas to provide context to their data and establish connections between metadata records and relevant files. To help researchers understand the connections and contextual relationships within their dataset, there are built-in visualizations for metadata graph structures. Metadata is also published alongside files in a dataset, allowing them to be integrated with other systems.

#### FAIR Data Sharing

To promote **findability**, Pennsieve assigns globally recognized DOIs to each published version of a dataset. These DOIs enable datasets to be cited and indexed through Google Dataset Search and other search engines for discovery by researchers worldwide. They also allow authors of datasets to receive credit when other’s cite their data. Pennsieve Discover is a public repository, closely linked to the platform, that makes published datasets freely **accessible** to the scientific community. Each dataset published through Pennsieve Discover has a dedicated page that includes details about the dataset and allows users to browse, visualize, and download its files (Fig. S8). Datasets are made **interoperable** by standardizing publishing schemas (the file structure of a dataset) and serializing all metadata into tabulated files. Lastly for **reusability**, Pennsieve’s data publishing mechanisms enforce strict quality standards that ensure datasets are well-documented and ready for reuse. All data, including serialized metadata tables, are exported to cloud object storage where users can fetch and interact with the data without platform restrictions or Pennsieve-specific tools.

Several large-scale, federally-funded programs leverage the Pennsieve platform to support data publication from their investigators. To facilitate high-quality data publications, Pennsieve has implemented several mechanisms to ensure that published datasets meet the requirements for FAIR sharing of data. These include: 1) required data fields such as tags, license, summary, description, and a contributor list, 2) processes for an external data curation team to review datasets prior to publication, and 3) procedures to submit and release new versions of datasets with a complete provenance history.

#### Data Reliability and Utilization

Data reliability on Pennsieve spans from initial upload to publication and continues through post-publication management. A manifest is created when files are uploaded to a dataset which details the upload status of each file and provides a check for dataset completeness. Once the files are uploaded, Pennsieve maintains a timestamped dataset activity log that attributes any modifications made to the corresponding user. This provides a transparent account of the dataset modification history. Pennsieve’s governance mechanisms allow administrators to control who can access, view, edit, or contribute to datasets. For published datasets, post-publication modifications are version controlled, with each dataset version assigned its own DOI. This allows researchers to reference specific versions of data in their work and enables other researchers to access the exact version of the dataset used in a particular study.

To ensure that published datasets are high-quality and FAIR-compliant, Pennsieve integrates both manual and automated review mechanisms into its data publishing workflow (Fig. S5). The process is designed to be flexible, accommodating the specific needs and standards of different scientific domains. Upon submission for publication, a dataset enters a structured peer-review pipeline where it is evaluated by a designated publishing team. This team, which can be configured for a specific consortium or workspace, manually assesses the scientific appropriateness, completeness, and quality of the data and metadata. This process is managed using status indicators that track a dataset’s progress through the curation workflow. A key part of this human-in-the-loop validation is providing iterative feedback; datasets that do not meet the quality standards can be returned to the authors for revision and resubmission. This review process is explicitly visualized in the platform’s publishing workflow, which includes steps for submission, evaluation by a peer-review panel, and final approval. These manual checks are complemented by automated validation routines. For example, the platform can incorporate domain-specific workflows, such as validators that check for compliance with the Brain Imaging Data Structure (BIDS) standard for neuroimaging data. Collectively, these quality control mechanisms ensure that datasets are thoroughly vetted and standardized before being made publicly available.

#### Data Integration and Standardization

Pennsieve standardizes data through a combination of protocols and tools that ensure consistency across datasets. The platform supports a range of standardized data formats and conducts validation and quality checks during upload and publication. Datasets are organized in a hierarchical structure that maintains a clear relationship between their different components. Published datasets adhere to a standardized directory structure with pre-defined dataset information fields. In addition to these structural protocols, the API can be used to implement standardized data processing and analysis steps.

#### Fostering Collaborative Science

**Fig. 2:**
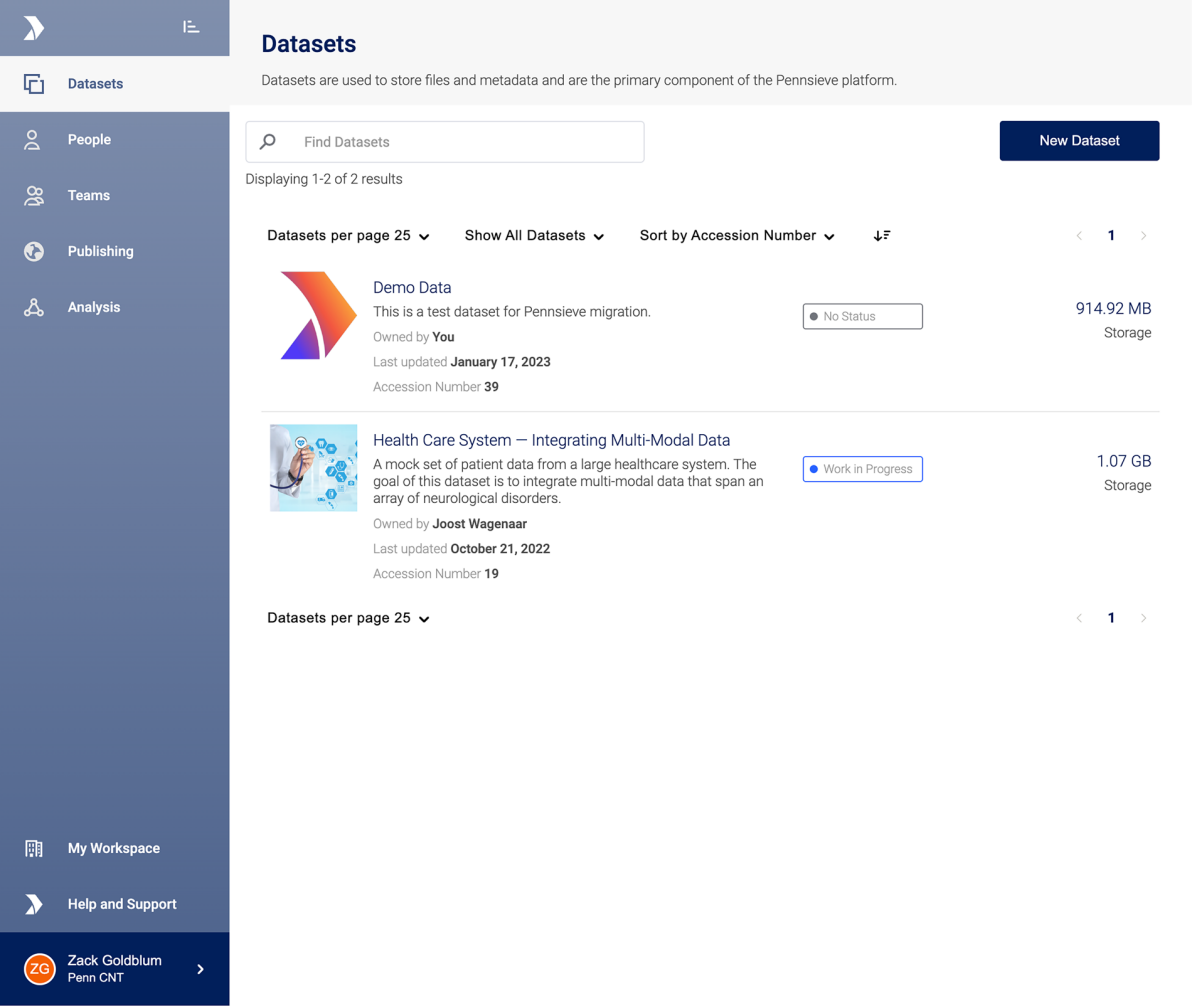
The Pennsieve platform’s web application interface. This workspace view highlights key features including dataset management, collaborative functionality, publishing workflows, and analysis tools.

The premise of the Pennsieve platform is to foster collaborative science, whether within a single lab, an interinstitutional consortium, or through public access of published datasets. Its multi-tenant architecture allows for independent workspaces that can be used by groups of researchers to organize, curate and privately share datasets (Fig. 2). Datasets are private by default; the owner of the dataset needs to specifically grant other users access to the dataset. Role-based permissions dictate the access and abilities of individual users or teams of users, and datasets can be shared with the entire workspace or only selected teams.

The platform is developed specifically for scientific use-cases and has dedicated functionalities for several data types, such as EEG, clinical imaging, and microscopy imaging (Fig. S7). It allows users to view and annotate these files directly in the web application and supports timeseries data streaming. These modality-specific features are key to making the platform more usable for collaborative research without relying on file sharing and external software packages.

#### Enabling Scalable Analysis

**Fig. 3:**
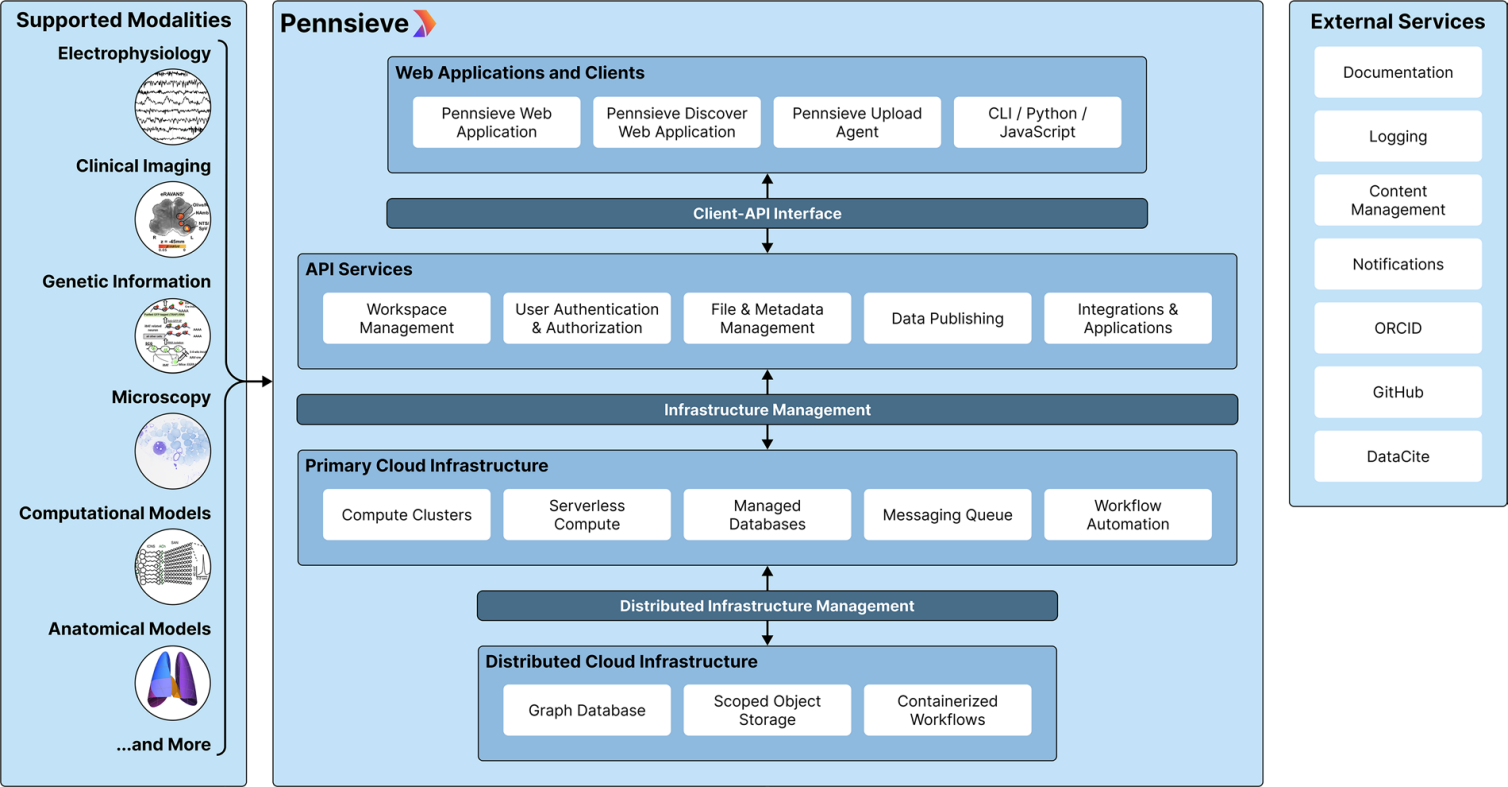
Technical overview of the Pennsieve platform. Its multi-tenant architecture provides private workspaces where research groups upload, analyze, and publish datasets while maintaining governance. Client applications interface with API microservices which scale to manage workspace-specific compute, storage, analysis workflows, and integrations with external tools (e.g., ORCID, GitHub, DataCite). Pennsieve operates all services on cloud infrastructure and allows users to attach their own compute and storage nodes to workspaces.

Pennsieve is architected as a collection of independent microservices deployed on Amazon Web Services (AWS) to ensure high availability, support for concurrent access, and large-scale dataset management (Fig. 3). To handle high-throughput requests from many simultaneous users, core API services are deployed on AWS Elastic Container Service (ECS) behind load balancers that use autoscaling to dynamically adjust to demand, with services distributed across multiple availability zones for fault tolerance. Newer services leverage a serverless architecture with AWS API Gateway and Lambda functions, providing automatic and massive scaling capacity. This hybrid approach ensures the platform can efficiently manage a high volume of simultaneous API requests and programmatic interactions.

The platform accommodates both a high number of files and very large individual files (up to 5 TB). In real-world use cases, researchers have successfully published datasets containing hundreds of thousands of individual files, as well as those with single files exceeding 100 GB. In these scenarios, the primary bottleneck for data transfer was the user’s local network bandwidth, not the platform’s backend infrastructure. This efficiency is achieved through a manifest-based data transfer workflow managed by the Pennsieve Agent. For both uploads and downloads, this process grants users secure, time-limited credentials to transfer data directly to and from AWS S3 cloud object storage. This method offloads the bandwidth and computational demands from Pennsieve’s servers directly to AWS’s highly scalable infrastructure. Recognizing that downloading petabyte-scale data is often impractical, Pennsieve supports in-cloud data analysis, allowing users to register their own compute resources to execute computational workflows directly on the hosted data, mitigating costs and overcoming the limitations of data transfer.

Pennsieve provides researchers with a suite of tools for managing and analyzing data at scale. Central to this is the Pennsieve Agent, a local application that manages large uploads and API interactions, which can be controlled via command-line interfaces in Python, Go, and JavaScript. The platform’s open API and use of webhooks further extend its capabilities by enabling integration with external applications and automated workflows. These tools enable the integration of user-created analytic pipelines that extend the platform’s utility beyond data storage. For example, the Center for Neuroengineering and Therapeutics (CNT) at the University of Pennsylvania utilizes Pennsieve to implement and execute standardized epilepsy co-registration pipelines, significantly reducing data analysis overhead across multiple clinical sites. In the field of immunology, the Penn Immune Health (I3H) project leverages Pennsieve to automate and scale their analytic workflows that generate reports for cell classification and immune profiling. The platform also supports externally developed applications, such as a webapp for normative modeling and visualization of iEEG data. These use cases highlight Pennsieve’s role as an active research environment where data is not only stored but also directly connected to scalable tools for analysis and discovery.

#### Resource Sustainability

Sustainability is a foundational principle of the Pennsieve platform, encompassing its technical architecture, data preservation policies, and financial model. The platform is designed to be a lasting resource that evolves with community needs while ensuring operational costs are managed effectively.

From a technical perspective, Pennsieve employs a cost-efficient architecture that balances elasticity with durability. Container-based services are combined with serverless workloads that scale to zero when idle, minimizing baseline spend while preserving on-demand computational capacity. The co-location of compute and data resources in the cloud improves the efficiency of in situ analysis. Datasets reside in AWS S3 under automated, tiered life-cycle policies that migrate inactive files from active storage to archive and deep-archive tiers, reducing costs without compromising durability. Pennsieve supports distributed data storage on a per-workspace basis, a capability leveraged by NIH programs through the STRIDES Initiative to manage data stores independently from the core platform. This also enables data to be stored in specific geographic regions to comply with local data sovereignty laws. For very large datasets (≥1 TB), mechanisms for requester-pays access and external funding via data sharing plans provide additional flexibility. Importantly, in the unlikely event that the platform itself can no longer be supported, Pennsieve’s data publishing model ensures permanent accessibility: all data and metadata are serialized into self-describing objects on AWS S3 during publication, enabling datasets to remain FAIR by redirecting DOIs to these files even without active platform infrastructure.

From a financial and institutional perspective, Pennsieve is supported by a hybrid strategy that combines grant funding, institutional commitments, and emerging cost-recovery mechanisms. Large-scale NIH initiatives, including the SPARC and HEAL programs, distribute operational costs and de-risk the platform from reliance on a single award. The University of Pennsylvania provides a critical layer of foundational support, committing to maintain submitted data for a minimum of 10 years regardless of active project funding. Several university institutes provide additional long-term financial backing. The strategic goal is to transition toward a self-sustaining, mixed-revenue model in which programs and users who derive value from the platform contribute to its maintenance. Current mechanisms include modest dataset submission fees (with publication of datasets under 25 GB remaining free of charge), while subscription and usage-based models are being evaluated to equitably distribute costs. To further diversify revenue streams, Pennsieve is pursuing HIPAA compliance attestation, which will enable support for clinical trials and unlock new, sustainable funding avenues.

Finally, Pennsieve’s open-source model and community engagement provide a practical layer of sustainability. The platform is openly available on GitHub, where developers can contribute code, report issues, and extend functionality through integrations with external tools. Detailed documentation, tutorials, and user guides support adoption and lower the barrier for community participation. In practice, this model has enabled contributions from both academic groups and NIH-funded consortia, ensuring that improvements to the platform are shared across projects rather than siloed. By aligning governance with transparency and reusability, Pennsieve maintains a development model that can persist beyond any single funding cycle.

### Impact

**Fig. 4:**
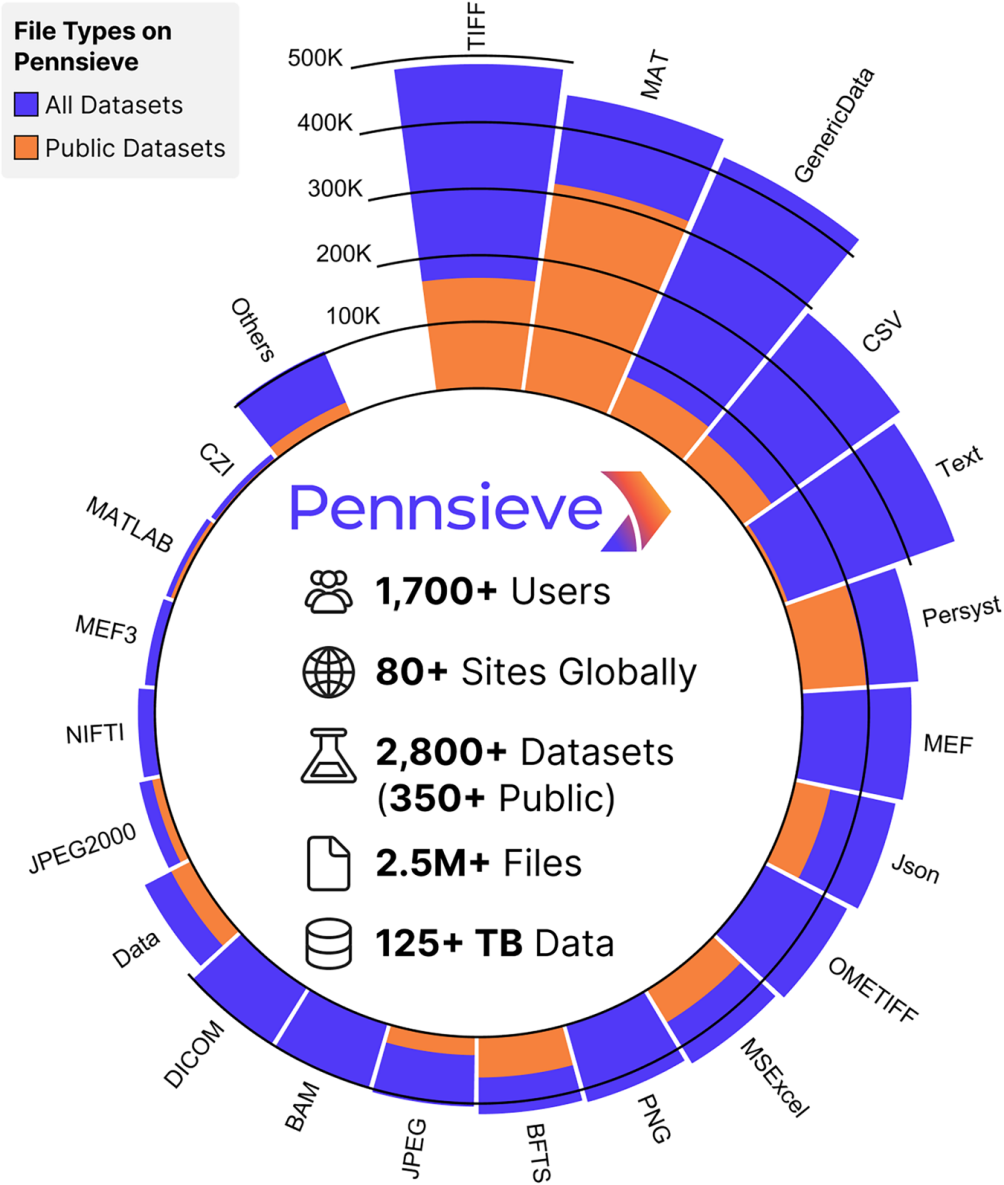
Pennsieve platform metrics and file type distribution. The circular chart illustrates the variety of file types supported, with comparative proportions for all datasets and public datasets.

Pennsieve supports research efforts ranging from individual investigator groups to large-scale, NIH-funded consortia. At the time of writing, Pennsieve has over 1,700 registered users from more than 80 research sites globally (Fig. S1). There are hundreds of daily users (Fig. S2) and on average, 80+ GB of data is downloaded weekly from its public services (Fig. S3). With more than 350 public datasets (2,800+ public and private combined), it stores over 2.5 million files comprising 125 TB of data (Fig. 4). These span from small collections (<10 GB) to large datasets (>5 TB) with complex data structures and metadata graphs. This makes Pennsieve one of the largest, fully-maintained data resources for the neuroscience community.

**Fig. 5:**
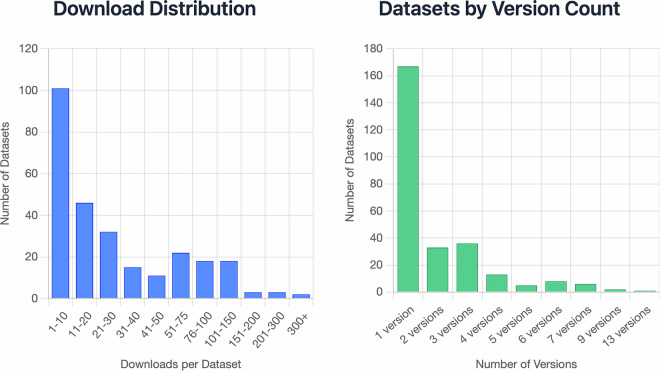
Dataset usage and versioning on Pennsieve. *Left:* Distribution of dataset downloads since 2023 (10,432 total downloads across 271 unique public datasets). *Right:* Distribution of the number of versions per dataset.

Since we began tracking download metrics in 2023, a total of 10,432 downloads have occurred across 271 unique public datasets. The distribution follows a characteristic long-tail pattern, with a small subset of datasets receiving disproportionately high engagement while the majority experience modest but consistent use (Fig. 5). These usage patterns highlight the platform’s role in both supporting widely reused community resources and enabling niche but valuable datasets to remain accessible.

#### Supporting Major Research Initiatives

In support of a collaborative research ecosystem, Pennsieve provides data management infrastructure for broader scientific repositories and projects. The includes several NIH initiatives: the Stimulating Peripheral Activity to Relieve Conditions (SPARC) program^50^, the Helping to End Addiction Long-term (HEAL) Initiative’s^51^ Restoring Joint Health and Function to Reduce Pain (RE-JOIN) Consortium, and the Program to Reveal and Evaluate Cells-to-gene Information that Specify Intricacies, Origins, and the Nature of Human Pain (PRECISION Human Pain) network. These initiatives, and more than 80 research groups worldwide, use Pennsieve for data submission, curation, and publication.

Pennsieve underpins several domain-specific portals that promote data accessibility in specialized research areas. The SPARC.Science portal serves as a central hub for datasets from the SPARC program, HEAL RE-JOIN Consortium, and HEAL PRECISION Human Pain network. It contains over 90 active projects, 220 datasets, 38 anatomical models, and 45 computational models focused on peripheral nervous system analysis. As a HEAL-compliant repository, it also accepts datasets from other HEAL initiatives and NIH efforts within the scope of pain and addiction research.

Pennsieve is also expanding its impact on epilepsy research through the Epilepsy.Science portal. This project brings together Pennsieve’s scalable data management, the Brain Data Science Platform (BDSP)’s extensive data resources and analytic tools^52^, and cloud credits from the Amazon Web Services (AWS) Open Data Sponsorship Program to sustain public access. The aim is to develop an extensive catalog of high-quality, multimodal epilepsy datasets from broad contexts for research and clinical translation.

Other specialized efforts are supported by Pennsieve. For example, the Pediatric Quantitative EEG Strategic Taskforce (PedQuEST), centered on advancing brain-focused care for critically ill children using quantitative EEG, and the Childhood Status Epilepticus and Epilepsy Determinants of Outcome (SEED) project, a extensive study on childhood status epilepticus in Nigeria.

The use of Pennsieve infrastructure to support these efforts demonstrates its utility beyond traditional neuroscience data management. Its capacity to handle large-scale, complex datasets makes it an impactful platform for various NIH programs and other targeted projects.

## Discussion

Pennsieve offers a comprehensive neuroscience data management platform that addresses many of the field’s current challenges. Its emphasis on metadata curation, collaboration, data publishing, and extensibility positions it well to support the demands of modern neuroscience research. As the field continues to generate increasingly large and complex datasets, platforms like Pennsieve will play an important role in catalyzing scientific insights in a variety of fields. Though current major efforts are focused on immune health and the brain and nervous system, Pennsieve’s reach is increasing daily and there is tremendous opportunity to leverage its open-source model in reaching other fields, applications, and disciplines.

Pennsieve’s unique approach focuses not only on making data publicly available but also on *how* datasets are presented and shared. Visually appealing and informative landing pages for public datasets aim to capture researchers’ interest and facilitate meaningful connections between data contributors and potential collaborators. Moreover, Pennsieve supports internal data sharing between collaborators within and across institutions. This lowers the barrier for researchers to make their data publicly available, aligning with open science initiatives and data sharing mandates.

Pennsieve’s transition from industry to academia was pivotal, as it reshaped the platform into a sustainability model that prioritizes open science and the FAIR principles. Consequently, Pennsieve gained credibility and adoption among researchers, with increased engagement from open-source initiatives. This transition from funded development to a sustainable operational model was challenging, and the constant effort to balance the immediate needs of supported projects with long-term sustainability considerations necessitated compromises. For instance, making large-scale data accessible at cost required a reduced focus on supporting analysis directly on the platform. Choices such as this reflect Pennsieve’s commitment to creating a sustainable infrastructure that can continue to serve both the neuroscience and broader scientific communities beyond initial funding phases.

### Future Directions

While Pennsieve offers comprehensive infrastructure for data management and collaboration, it is still in active development, with a roadmap for new functionality spanning the coming years. As part of this roadmap, we will pilot modest, usage-based fees and hybrid funding models to keep long-term operating costs sustainable. We are increasingly focused on supporting distributed analytics through the platform, and expect that these capabilities will increase Pennsieve’s value to researchers beyond data management and publication. In addition, we are actively pursuing integrations with other platforms - both academic and commercial - to expand Pennsieve’s impact within the larger ecosystem of scientific tools and infrastructure available to the neuroscience community. We strongly believe that the next 10 years of neuroscience research will trend towards the integration of analytic tools, repositories, and other efforts that systematically increase our ability to leverage data for new discoveries and the development of cures for patients with disease.

Our vision for Pennsieve is inextricably tied to the evolution of data platforms and utilities across multiple sectors. As science, business, education, and government grapple with exponentially expanding data, platforms like ours promise to address some of society’s most difficult problems. Sustainability, as mentioned earlier, remains a key concern. We firmly believe that our platform and others will remain self-sustaining if they continue to provide real value to their users.

At the same time, it is vital to acknowledge that no single platform will dominate this space. The future lies in linking platforms across groups and fields, and sharing the most valuable tools between them. Several challenges persist in this domain: addressing the “data lifecycle”, navigating privacy concerns and data de-identification, balancing data sharing across institutions while maintaining individual control and ownership of resources, and reconciling the revenue focus of for-profit entities with societal needs to leverage data, reduce costs, and improve the human condition.

These challenges are not unique to data platforms and sharing in the biological sciences; they extend to many activities affecting our daily lives. While the ideas presented here are a start to this process, we anticipate more complex discussions as relevant technologies advance in complexity and societal uptake increases.

## Supplementary information

Supplementary Material

## Data Availability

All datasets supporting this manuscript are hosted on the Pennsieve Discover platform: https://discover.pennsieve.io. Public datasets can be accessed through their respective DOIs, which are listed in the manuscript or its Supplementary Material.

## Code availability

The Pennsieve platform and its services are open source and available on GitHub: https://github.com/Pennsieve. Documentation for using the Pennsieve API, programmatic upload/download tools, and example workflows in Python, JavaScript, and other supported languages can be found in the Pennsieve Documentation Hub (https://docs.pennsieve.io/) and in their respective GitHub repositories.
