# Supplementary material for: Pennsieve: A Collaborative Platform for Translational Neuroscience and Beyond
Source: Sci Data. 2025 Nov 19;12:1834. doi: 10.1038/s41597-025-06075-5 (PMC12630885; doi:10.1038/s41597-025-06075-5)
Supplement: Supplementary file 1 — Supplementary Material [file 41597_2025_6075_MOESM1_ESM.pdf]

Supplementary Material

Pennsieve Platform

To access the platform, visit ***Pennsieve***. For public datasets, explore ***Pennsieve Discover***. If you would like to publish your data on Pennsieve, please reference the **publishing documentation**. Extensive documentation for Pennsieve can be found in the **Documentation Hub** and the **API reference** provides code snippets and tutorials for interacting with the Pennsieve API in more than 20 programming languages. The fully open-source code for the platform can be found on ***GitHub***.

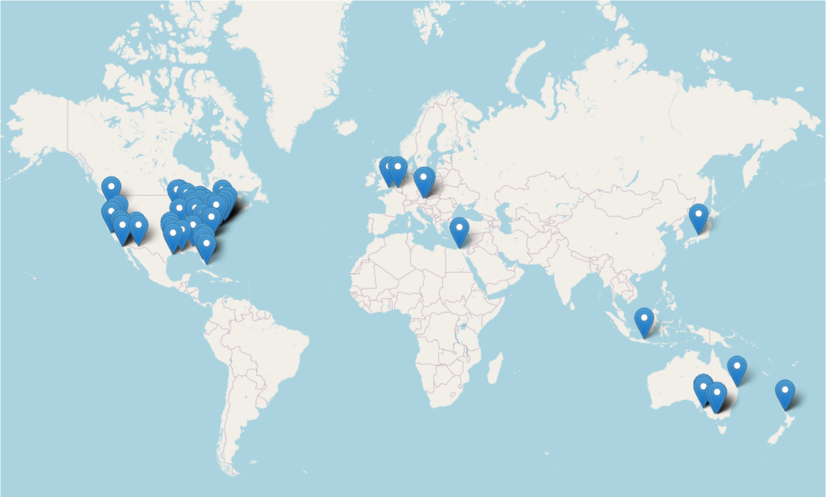

Figure S1: Pennsieve is utilized by more than 1,700 users across 80+ research sites worldwide.

Data Management Workflow

Pennsieve supports the life cycle of scientific data from initial upload through publication. The user workflow, detailed in Fig. S4, encompasses several key functionalities: secure data upload, dataset management, collaboration within workspaces, dataset curation, and dataset publishing.

1. Pennsieve supports file uploads through both its web application and programmatic methods. The upload process involves three steps: 1) generation of an upload manifest, 2) uploading the files, and 3) verification of the uploaded manifest. Large datasets should be uploaded programmatically to the platform and the Pennsieve Agent - an installable application for Windows, macOS, and Linux - facilitates this process. The agent manages manifest generation and file uploading to the platform. Several tools are

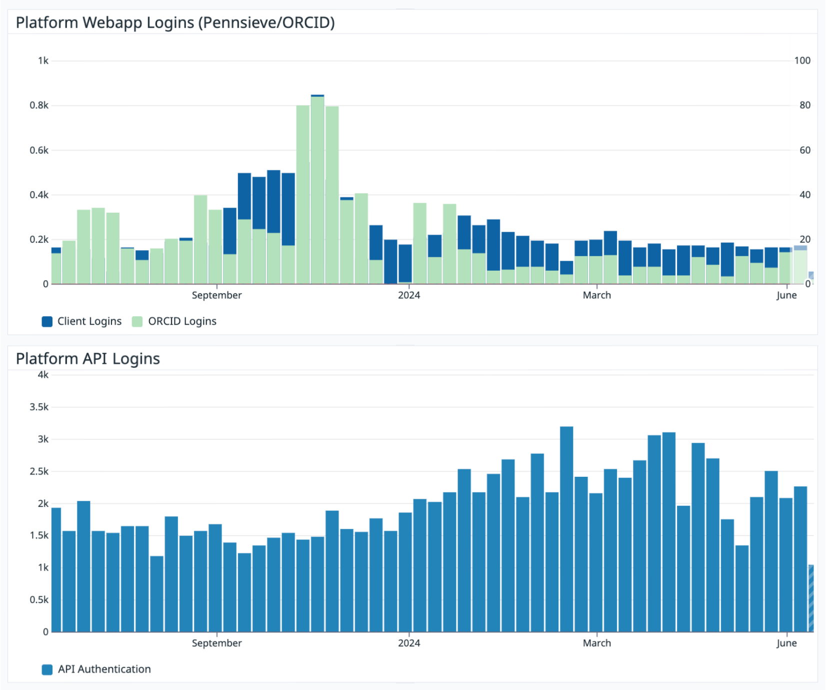

Figure S2: Daily user engagement with Pennsieve. *Top:* Web application logins, distinguishing between standard user logins and ORCID authentications. *Bottom:* API authentication requests, indicating programmatic platform usage.

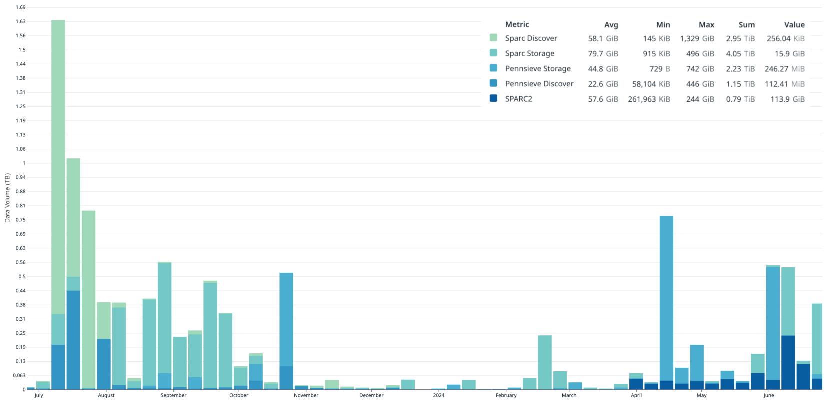

Figure S3: Monthly data volume (in TB) downloaded from Pennsieve and related services. The stacked bar chart shows the distribution across different services. Public services (Pennsieve Discover and SPARC Discover) account for a significant portion of the transfers.

available to interact with the agent, including a Python client, CLI tool, and Go library. The agent exposes a gRPC interface which makes it easy for users to develop integrations in other programming languages. Prior to uploading files, the manifest is synchronized with the cloud, enabling users to track expected data uploads over time and resume uploads in case of a client disconnection.

2. Data on Pennsieve are stored using AWS S3 cloud object storage and made available to users in the form of datasets, the primary components of Pennsieve. Datasets have a directory structure and actions such as renaming, moving, downloading, or deleting files and folders can be done through the web interface or programmatically via the Pennsieve API. Data file types are automatically analyzed and when applicable, converted into proprietary packages that enable direct interactions with the data on Pennsieve. Additional functionality is available for such files, and this derived data is stored alongside the original files. Metrics including total number of files, dataset size, and last updated date are available in an overview pane that provides a comprehensive summary of the dataset. Numerous dataset attributes can be specified: dataset name, subtitle, contributors, description, license, tags, and banner image. Users can keep datasets private or choose to share them with collaborators or entire workspaces on Pennsieve.

3. Workspaces are shared environments that users can create, or join, where datasets and tools can be collectively utilized. In these workspaces, users can share datasets, add collaborators, manage user permissions through role-based access controls, and organize into teams. This framework provides control over who can view, edit, and manage specific datasets. Discussion functionality integrated within timeseries and imaging viewers lets users collaborate alongside their visualized data.

4. The platform provides a set tools to associate complex metadata with datasets and files. Users can define a metadata schema for each dataset and create records linking to files. For example, a user could define a metadata schema of "Patients", "Hospital Visits", and "Samples", which are all linked and point to specific files. Or a different schema could be defined with "Animal", "Experiment", and "Trial" to capture a very different type of scientific dataset. This lets researchers define cohorts within their datasets, allowing for targeted studies and analyses. This capability is particularly useful for clinical and biomedical research, where grouping data by specific characteristics (e.g., patient demographics or treatment groups) can provide valuable insights. The outcome of this continuous curation process is high-quality datasets suitable for reproducible scientific research.

5. The final stage of the workflow is publishing datasets to public repositories, making them accessible, citable, and reusable by the scientific community. The data publishing process is fully detailed in the following section.

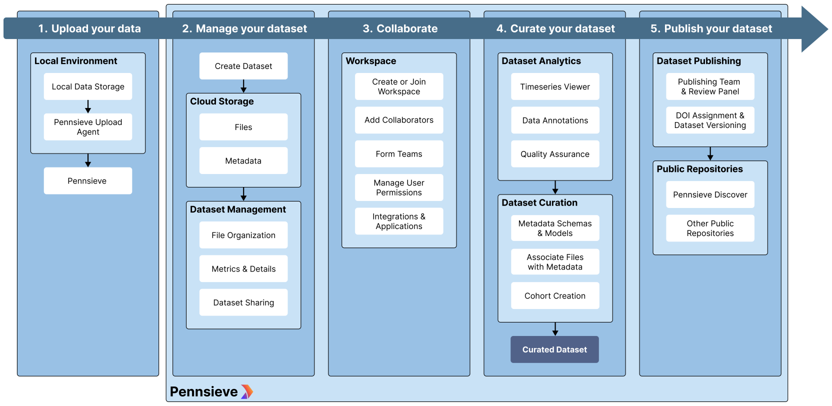

Figure S4: Pennsieve's end-to-end data management workflow. The platform supports the full lifecycle of scientific data, from initial upload to final publication in public repositories.

Data Publishing

The data publishing process on Pennsieve (Fig. S5) is how curated datasets get approved and finalized for distribution in public repositories. When a dataset reaches a publication-ready stage, the owner of the dataset can submit a request in their workspace for dataset peer-review. A publishing team, comprised of selected users with owner or administrator privileges, receives this request for evaluation. If the dataset is rejected, it can be revised for re-submission, while accepted datasets proceed in the publication process. A DOI is assigned to the dataset and it becomes version controlled. An optional embargo period of up to one year can be applied, giving authors control over when their dataset becomes publicly accessible. Finally, once all requisite steps are completed, the dataset is published in the chosen public repository. Any changes published after this point are controlled, with the modified dataset receiving a new version number and DOI.

Documentation Guides, Tutorials, and Recipes

Multiple guides, tutorials, and runnable code snippets are available in the ***Pennsieve Documentation Hub***. This includes instructions for how to set up accounts, workspaces, and manage datasets from upload through publication. ***Recipes*** - manuals which break down source code into explainable chunks - detail common interactions with the platform.

Pennsieve API Reference

The ***API Reference*** provides detailed information on the available API calls that allow for extensive interactions with the platform.

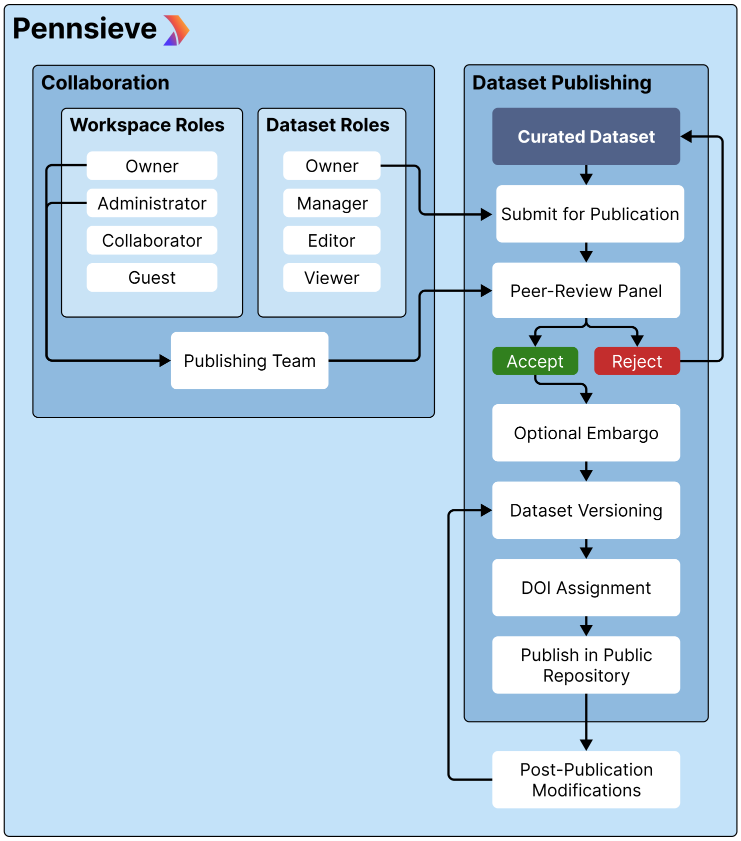

Figure S5: Pennsieve's data publishing workflow. The progression from curated dataset to public repository includes peer review, version control, and DOI assignment.

Open-Source Code and Microservices

Pennsieve is fully open-source and built with a microservices architecture. The ***Pennsieve codebase*** is deployed to the AWS cloud in an Infrastructure as a Service (IaaS) model using Terraform and Jenkins. It is built as a collection of independent microservices that exchange information with each other and provide a uniform experience to the user. Here are the primary Pennsieve code repositories:

- ***Authentication Service*** - Manages user and data permissions using AWS Cognito (CIAM).
- ***Pennsieve App*** and ***Pennsieve Discover App*** - Manage web applications and content visible to the users.
- ***Pennsieve API*** and ***Pennsieve Go API*** - Allow interaction with Pennsieve through its API.
- ***Discover Service*** - Manages the collection of public datasets.
- ***DOI Service*** - Creates, manages, and publishes DataCite DOIs.
- ***Model Service*** and ***Serverless Model Service*** - Provide a graph layer to the data (metadata and annotations).
- ***Discover Publish***, ***Publishing Service***, and ***Datasets Service*** - Handle publishing and managing datasets.
- ***Discover Release*** - Manages embargoed and private datasets.
- ***Pennsieve Agent***, ***Pennsieve Agent Python***, and ***Pennsieve Agent JavaScript*** - Interfaces in Go, Python, and JavaScript that interact with the platform and allow users to programmatically upload and download files.
- ***Workflow Manager***, ***ETL Nextflow***, and ***App Deploy Service*** - Create, orchestrate, and manage applications and workflows on Pennsieve.
- ***Rehydration Service*** - Retrieves datasets or files from any of the previously released versions and prepares them for download.
- ***Integration Service*** - Creates webhooks and manages integrations and notifications.
- ***Timeseries Processor*** - Ingests data to Pennsieve and stream, download, and display data.
- ***App Deploy Service*** - Sets up infrastructure in AWS.
- ***Upload Service*** - Uploads large files to Pennsieve.

Data Security

Built on AWS S3, Pennsieve utilizes cloud infrastructure to provide high scalability, security, and availability for file storage. Individual files up to 5TB in size are supported and an essentially unlimited number of files can be handled. AWS S3 storage provides 99.999999999% (11 nines) annual durability protection from loss or corruption and 99.99% (4 nines) availability. All uploaded files undergo checksum testing and are secured at rest using server-side encryption (SSE). Data transfers on Pennsieve are secured with Secure Sockets Layer (SSL) encryption to protect it from interception and unauthorized access. Pennsieve supports encryption keys on a per-workspace basis and distributed data storage to further compartmentalize access if necessary for security or compliance reasons. These systems safely and reliably persist data at scale.

Governance and Sustainability

Pennsieve's governance structure has several mechanisms that ensure its sustainability, compliance with standards, and responsiveness to user needs.

Advisory Boards

Three advisory boards guide Pennsieve:

1. Clinical Advisory Board: Provides guidance on data standards, clinical workflows, and how to enhance the platform's impact on patient care. This advisory board is currently being established in step with the platform's increasing application in clinical research.

2. Technical Advisory Board: Advises on implementing technical standards, developing the platform's roadmap, and integrating with other data science efforts.

3. Team Blue: Prioritizes non-critical feature requests and provides user feedback that aligns Pennsieve's development with user needs.

The technical and clinical advisory boards meet biannually to steer Pennsieve's strategic direction and include representatives from academia, non-profit organizations, and industry partners. They are responsible for reviewing and approving policies related to the platform, including data submission criteria and standards for dataset inclusion. Team Blue is a user-based advisory panel that convenes every two months and is comprised of at least four external and two Penn-based investigators actively using Pennsieve.

Compliance and Certifications

Pennsieve meets all required and recommended aspects of FAIR data sharing and is in the process of obtaining the CoreTrustSeal certification. Additionally, Pennsieve is GDPR compliant and is developed with HIPAA-certification in

mind. Currently, it does not claim HIPAA compliance and does not accept PHI data. The Pennsieve team is working with the University of Pennsylvania towards HIPAA compliance attestation.

How can I contribute?

Pennsieve is committed to high standards of data quality and encourages members of the scientific community to contribute their data to the platform.

Researcher

Pennsieve serves researchers as a platform for publishing, sharing, and annotating data. Documentation on setting up an account can be found in the ***Getting Started with Pennsieve*** tutorial. Researchers may upload their files to the platform using the Pennsieve Agent (see ***tutorial***). This is the recommended method for large or complex datasets. The Pennsieve Agent is a local, lightweight client that controls and verifies data transfer to cloud storage in Pennsieve. It allows users to prepare files for upload in a package and monitors the upload status. Currently, Python, JavaScript, and CLI clients are supported on all major platforms (Windows/macOS/Linux).

Researchers can use several features on Pennsieve to create and contribute datasets. ***Collaborative workspaces*** are shared environments where datasets and tools can be utilized collectively. Researchers can manage user permissions through role-based access controls and organize their members into teams. ***Dataset curation*** tools allow direct data and metadata management. ***Publication pipelines*** enable publishing to public repositories, making datasets accessible, citable, and reusable by the scientific community.

Data Analyst

Pennsieve streamlines downloading and managing data by providing a standard, open API. Requests to and responses from the server can be easily tested in 20 different programming languages; please refer to the ***Pennsieve API Reference*** for more details.

Downloading publicly available files is straightforward using the Pennsieve Agent. Examples for querying and obtaining data from Pennsieve can be found in the ***NIH SPARC Python Client Tutorial***. Documentation on its functions is available in the ***Package Reference***.

Data analysts are encouraged to share their analytic workflows. By contributing scripts, pipelines, and methodologies to the Pennsieve community, data analysts support reproducible research.

Developer

Developers can contribute to Pennsieve's open-source codebase by fixing bugs, adding features, or improving documentation. The Pennsieve API can be used

to add new endpoints, improve existing ones, or add integrations with other tools and platforms. The ***serverless service template*** details how developers can create microservices that extend Pennsieve's capabilities to offer new tools to the community.

Data Annotator

Pennsieve provides user-friendly tools for annotating ***timeseries*** and ***clinical imaging*** data. These annotations can be layered over the data, adding additional context and insights. Annotators can create relationships between metadata models and link records to specific files, creating a structured, searchable dataset. A graph metadata viewer makes it easier to understand complex datasets by visualizing the relationships between data and metadata.

User

Users have free access to data through ***Pennsieve Discover***. They can browse and download publicly available datasets from a range of scientific fields. Each dataset includes the metadata, annotations, and related documents necessary for its reuse. Search tools help users find datasets relevant to their research interests, with options to filter results by tags and dataset status. Additionally, they can engage with the community by participating in ***discussions*** and initiating collaborations with dataset authors.

Datasets on Pennsieve

Dataset Diversity

Pennsieve hosts over 350 publicly available datasets. These datasets span many neuroscience topics: vagus nerve stimulation (VNS), neural circuits, synaptic activity, electrophysiology, neurotransmitter systems, neural devices, neural imaging, computational models, gene expression, neural development, plasticity, and neural injury and repair. They provide a comprehensive resource for the neuroscience research community across multiple modalities including EEG, MEG, MRI, microscopy images, gene data, 3D models, and videos. Highlights of some datasets hosted on Pennsieve are presented in Fig. S6.

Data Viewing and Annotations

In scientific data analysis, annotations are crucial to the data themselves. Recognizing this, Pennsieve has developed viewers for various data modalities that allow users to view both data and annotations directly on the platform. These data viewers feature integrated tools for adding new annotations to datasets as overlaying layers. Fig. S7 provides examples of these viewers and annotation tools. More details about the viewers can be found in the ***documentation***.

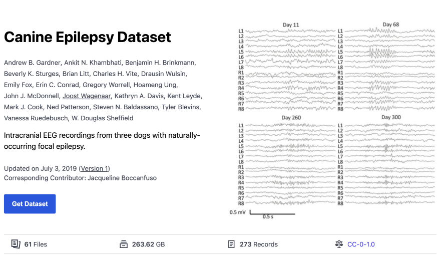

(a) Electrophysiology [1].

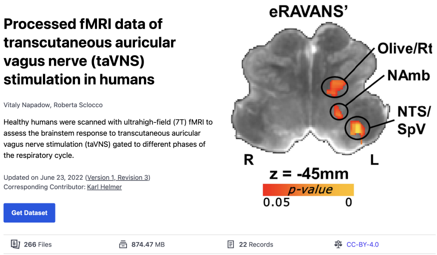

(b) Clinical imaging [2].

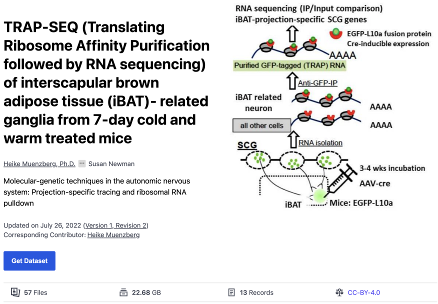

(c) Genetic information [3].

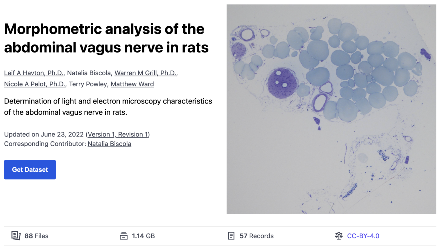

(d) Microscopy [4].

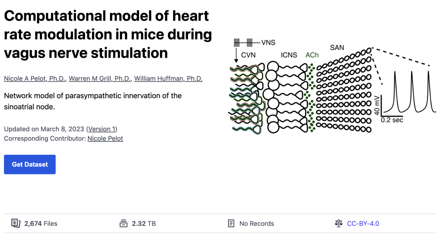

(e) Computational models [5].

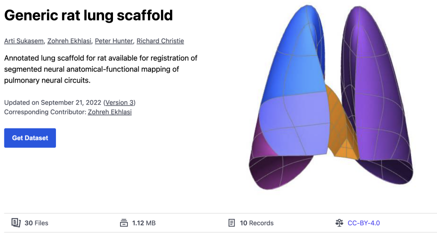

(f) Anatomical models [6].

Figure S6: Selection of diverse neuroscience datasets published on Pennsieve.

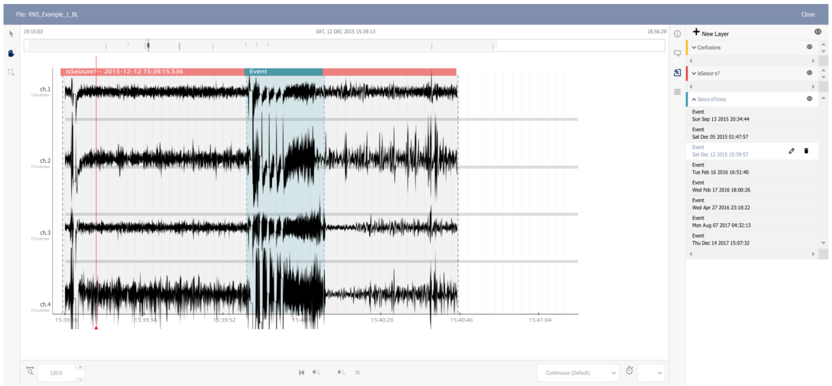

(a) Timeseries viewer with annotations.

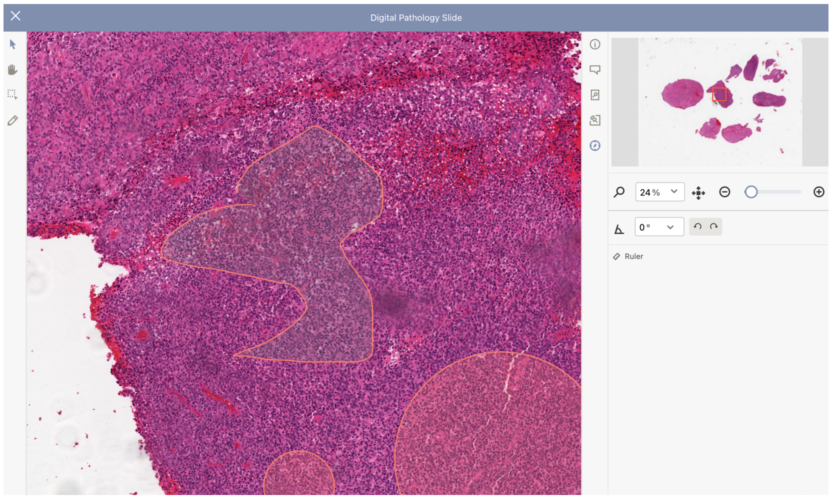

(b) Imaging viewer with annotations.

Figure S7: Integrated data viewers with annotation tools on Pennsieve.

Dataset Structure

Pennsieve Discover organizes each dataset page with a user-centric design, ensuring researchers can easily access its content and understand the scope and relevance to their own work. An example dataset page on Pennsieve Discover is shown in Fig. S8. The entire dataset, or individual files from it, can be downloaded directly from its page. The key sections include:

- Header
  - Title: Name of the dataset.
  - Contributors: Individuals who contributed to the dataset, along with their affiliations and roles.
  - Description: A brief summary of the dataset.
- Metrics
  - Number of files, dataset size, number of metadata records, and the license.
- Dataset Overview
  - Study Purpose: Describes the scientific objective and background of the study.
  - Data Collection: Details the methods and protocols used to gather the data.
  - Primary Conclusion: Summarizes the main findings and significance of the dataset.
- Curator's Notes
  - Experimental Design: Specifies whether the study is experimental, observational, or computational.
  - Completeness: Indicates whether the dataset is complete or ongoing.
  - Subjects & Samples: Provides details on the subjects or samples used in the study, if applicable.
  - Primary vs Derivative Data: Clarifies whether the dataset contains primary data, derivative data, or both.
  - Code Availability: Links to any code or models associated with the dataset.
- Files
  - Displays a directory structure of the dataset, including data files, metadata, and any additional documents such as readme files or changelogs.
- About

– Publishing History: Dates of original publication and last modification, also specifying the current version of the dataset.
– Citation & Sharing: The DOI for the dataset, the appropriate citation in several formats, and shortcuts to share the dataset on social platforms.
– Tags: Relevant keywords and tags associated with the dataset for easier discovery and categorization.
– References: Citations of literature that utilize this dataset.

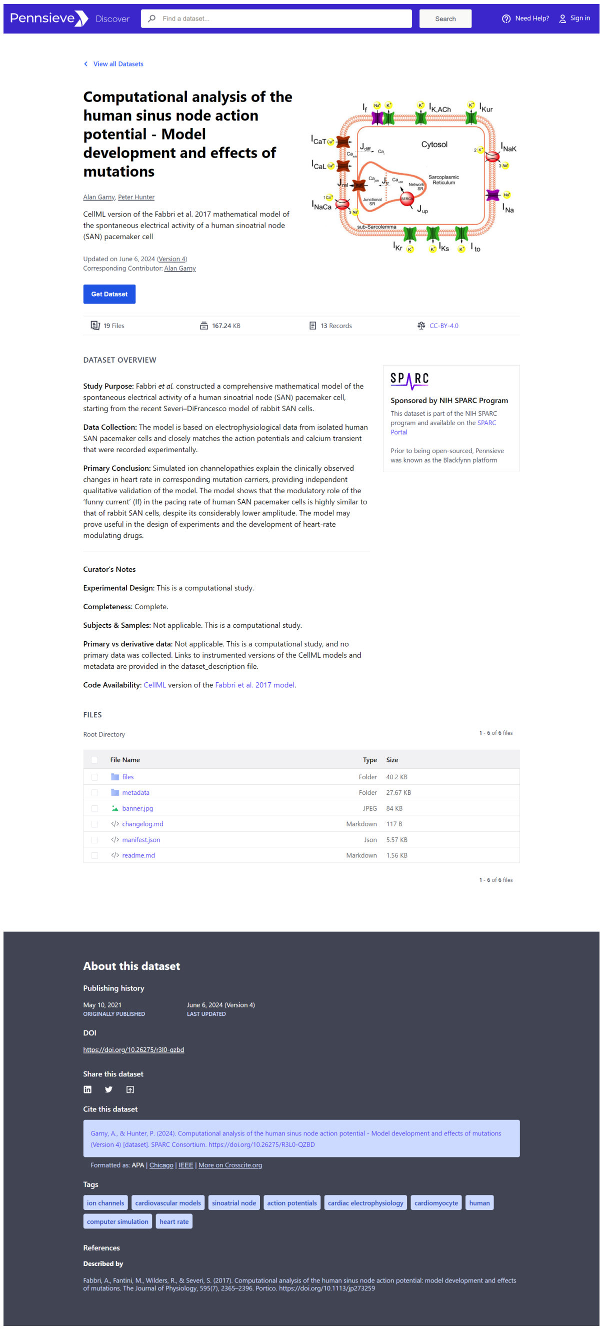

Pennsieve Discover | Find a dataset... | Search | Need Help? | Sign in

View all Datasets

Computational analysis of the human sinus node action potential - Model development and effects of mutations

Alan Garny, Peter Hunter

CellML version of the Fabbri et al. 2017 mathematical model of the spontaneous electrical activity of a human sinoatrial node (SAN) pacemaker cell

Updated on June 6, 2024 (Version 4)
Corresponding Contributor: Alan Garny

Get Dataset

19 Files | 167.24 KB | 13 Records | CC-BY-4.0

DATASET OVERVIEW

Study Purpose: Fabbri *et al.* constructed a comprehensive mathematical model of the spontaneous electrical activity of a human sinoatrial node (SAN) pacemaker cell, starting from the recent Severi–DiFrancesco model of rabbit SAN cells.

Data Collection: The model is based on electrophysiological data from isolated human SAN pacemaker cells and closely matches the action potentials and calcium transient that were recorded experimentally.

Primary Conclusion: Simulated ion channelopathies explain the clinically observed changes in heart rate in corresponding mutation carriers, providing independent qualitative validation of the model. The model shows that the modulatory role of the 'funny current' (If) in the pacing rate of human SAN pacemaker cells is highly similar to that of rabbit SAN cells, despite its considerably lower amplitude. The model may prove useful in the design of experiments and the development of heart-rate modulating drugs.

SPARC

Sponsored by NIH SPARC Program

This dataset is part of the NIH SPARC program and available on the SPARC Portal

Prior to being open-sourced, Pennsieve was known as the Blackfynn platform

Curator's Notes

Experimental Design: This is a computational study.

Completeness: Complete.

Subjects & Samples: Not applicable. This is a computational study.

Primary vs derivative data: Not applicable. This is a computational study, and no primary data was collected. Links to instrumented versions of the CellML models and metadata are provided in the dataset_description file.

Code Availability: CellML version of the Fabbri et al. 2017 model.

FILES

Root Directory | 1 - 6 of 6 files

File Name	Type	Size
files	Folder	40.2 KB
metadata	Folder	27.67 KB
banner.jpg	JPEG	84 KB
changelog.md	Markdown	117 B
manifest.json	Json	5.57 KB
readme.md	Markdown	1.56 KB

1 - 6 of 6 files

About this dataset

Publishing history

May 10, 2021 — ORIGINALLY PUBLISHED
June 6, 2024 (Version 4) — LAST UPDATED

DOI

https://doi.org/10.26275/r3l0-qzbd

Share this dataset

Cite this dataset

Garny, A., & Hunter, P. (2024). Computational analysis of the human sinus node action potential - Model development and effects of mutations (Version 4) [dataset]. SPARC Consortium. https://doi.org/10.26275/R3L0-QZBD

Formatted as: APA | Chicago | IEEE | More on Crosscite.org

Tags

ion channels | cardiovascular models | sinoatrial node | action potentials | cardiac electrophysiology | cardiomyocyte | human | computer simulation | heart rate

References

Described by

Fabbri, A., Fantini, M., Wilders, R., & Severi, S. (2017). Computational analysis of the human sinus node action potential: model development and effects of mutations. The Journal of Physiology, 595(7), 2365–2396. Portico. https://doi.org/10.1113/jp273259

Figure S8: Example of a published dataset page on Pennsieve Discover.

Pennsieve's Current Data Repository

Table S1: Public datasets available on Pennsieve (as of July 3, 2024)

DOI	Owner	Title	Year
10.26275/b0lj-xsto	Adeodu, O.	Simulation of the short term impact of atrial fibrillation on hemodynamic variables	2024
10.26275/qach-onkl	Keast, J.	Selective recording of physiologically evoked neural activity in a mixed autonomic nerve using a minimally invasive array	2024
10.26275/yues-hayo	Garny, A.	Fabbri-based composite SAN model	2024
10.26275/nbex-w34w	Garny, A.	Computational analysis of the human sinus node action potential - Model development and effects of mutations	2024
10.26275/mgyp-rlmr	Pelot, N.	Excitation properties of computational models of unmyelinated peripheral axons	2024
10.26275/yssz-iprs	Lin, M.	Generic human stomach scaffold	2024
10.26275/s3l0-t915	Lin, M.	A 3D human whole-body model with integrated organs vasculature musculoskeletal and nervous systems for mapping nerves	2024
10.26275/t2ve-vu9o	Aguinaga, D.	Targeting bladder function with network-specific epidural stimulation after chronic spinal cord injury	2024
10.26275/mu8z-s80h	Arendt-Tranholm, A.	Long read sequencing of human dorsal root ganglia	2024
10.26275/rdr5-x5bo	Ardell, J.	Transcriptomic and neurochemical analysis of the stellate ganglia in mice highlights sex differences	2024

DOI	Owner	Title	Year
10.26275/odx3-c5cv	Stebbing, M.	Quantification of enteric ganglia in the three main regions of the rat stomach	2024
10.26275/3enb-ctj6	Jaffey, D.	Vagal preganglionic axons arborize in myenteric plexus into two patterns generating nitrergic and non-nitrergic postganglionic motor units	2024
10.26275/hipp-yfnn	Wang, L.	Vasculature in mouse colon and relationship with enteric nervous system glia and macrophages	2024
10.26275/xw1e-oea3	Keast, J.	Light microscopic analysis of synaptic input to neurons in the rat major pelvic ganglion	2024
10.26275/pc5x-w5l3	Wang, L.	Transduction of systemically administered adeno-associated virus in the colonic enteric nervous system of adult mice	2024
10.26275/1ito-vvqo	Nguyen, D.	Substance P-immunoreactive axons in the antrum-pylorus-duodenum of mice	2024
10.26275/cheq-otb2	Wang, L.	Intravenously injected AAV9 transduced interstitial cells of Cajal in mouse colon	2024
10.26275/cljd-yjqy	Nguyen, D.	Calcitonin gene-related peptide - immunoreactive (CGRP-IR) axon innervation of mouse stomach	2024
10.26275/zphj-6qnb	Taylor-Clark, T.	Mapping of vagal sensory nerve populations and their brainstem projections in mice	2024
10.26275/aw2z-a49z	Armitage, O.	Influence of vagus nerve stimulation on vagal and cardiac activity in freely moving pigs	2024

DOI	Owner	Title	Year
10.26275/ixhd-0pfa	Athavale, O.	A mathematical model for simulating the neural regulation of phasic contractions and slow waves in the distal stomach	2024
10.26275/2njc-khrp	Venkataraman, A.	Effects of manipulating epithelial cell-nerve signaling on anxiety-like behavior in mice	2024
10.26275/2xys-dleh	Pelot, N.	Electromyogram recordings in mice during vagus nerve stimulation	2024
10.26275/eauv-gxwl	Pelot, N.	Electrocardiogram recordings in mice during vagus nerve stimulation	2024
10.26275/q2w0-keol	Williams, A.	Two-Color Diffuse In Vivo Flow Cytometer	2024
10.26275/86ve-meck	Bendowski, K.	Distribution and morphology of calcitonin gene-related peptide (CGRP) innervation in flat mounts of whole rat atria and ventricles	2024
10.26275/lcqj-dvpi	Ardell, J.	Sex differences in sympathetic gene expression and cardiac neurochemistry in Wistar Kyoto rats	2024
10.26275/gud3-alhy	Stebbing, M.	Quantitation of nodose neurons labelled by retrograde tracing from selective gastric mucosal and gastric muscle injection	2024
10.26275/n12j-ubmj	Stebbing, M.	RNAseq analysis of nodose neurons projecting to specific regions and tissue layers within the rat stomach	2024
10.26275/79vx-xnat	Stebbing, M.	Quantitation of nodose neurons labelled by retrograde tracing from selective injections into the gastric mucosa or muscle in the antrum and fundus	2024

DOI	Owner	Title	Year
10.26275/yv7l-9fst	Howard, M.	Synaptic components function and modulation characterized by GCaMP6f calcium imaging in cholinergic myenteric ganglion neurons	2024
10.26275/tlgo-dfke	Stebbing, M.	Quantification of thickness of the gastric muscle in the rat stomach	2024
10.26275/mhji-cn6n	Stebbing, M.	Morphologies dimensions and targets of gastric nitric oxide synthase neurons in the rat stomach	2024
10.26275/3d2g-d3xd	Iavarone, E.	ASCENT Tutorial	2023
10.26275/0jz3-zrlo	Iavarone, E.	ASCENT Guided Mode Demo	2023
10.26275/yanw-4h7n	Iavarone, E.	ASCENT Base	2023
10.26275/qdv4-0vx5	Bizanti, A.	Chronic intermittent hypoxia remodels catecholaminergic innervation in mouse atria	2023
10.26275/pmnr-f6br	Stacey, W.	Michigan High Res EEG - UMHS0018	2023
10.26275/jkbj-f3bn	Stacey, W.	Michigan High Res EEG - UMHS 0028	2023
10.26275/s3jn-eo59	Stacey, W.	Michigan High Res EEG - UMHS 0023	2023
10.26275/73j6-8nej	Stacey, W.	Michigan High Res EEG - UMHS 0022	2023
10.26275/ngob-yc3c	Stacey, W.	Michigan High Res EEG - UMHS 0019	2023
10.26275/hhnz-div3	Taylor-Clark, T.	Mapping of dorsal root ganglia sensory nerve populations in the mouse lung	2023
10.26275/jh8n-lwdl	Danziger, Z.	Reflex voiding in rat occurs at consistent bladder volume regardless of pressure or infusion rate	2023

DOI	Owner	Title	Year
10.26275/ile7-wrsk	Bedard, P.	Deep phenotyping of Post-infectious Myalgic Encephalomyelitis-Chronic Fatigue Syndrome	2023
10.26275/eefp-azay	Biscola, N.	High-throughput segmentation of rat unmyelinated axons by deep learning	2023
10.26275/4p8l-n5kt	Pattnaik, A.	iEEG networks preprocessing study	2023
10.26275/uz3t-mppx	Patel, B.	Fecobionics study in fecal incontinence (FI) human subjects	2023
10.26275/ahqc-ldar	Verheyden, J.	Identification of lung innervating sensory neurons and their target specificity in mouse (3)	2023
10.26275/sp7z-ylun	Moss, A.	Molecular phenotype distribution of single rat intracardiac neurons	2023
10.26275/qonw-sqix	Musselman, E.	Computational model of vagus nerve stimulation in mice with bipolar cuff electrode	2023
10.26275/7mdx-asxc	Pelot, N.	Histology-based computational models of implanted human cervical vagus nerve stimulation with the LivaNova helical cuff electrode	2023
10.26275/df7j-e48n	Musselman, E.	Pig-specific computational models of monopolar vagus nerve stimulation with a six-contact cuff electrode	2023
10.26275/wglu-jiud	Pelot, N.	Validated computational models predict vagus nerve stimulation thresholds in preclinical animals and humans	2023
10.26275/uco6-ktjq	Tohara, K.	Mouse genetic models to manipulate enterochromaffin cell activity - Murine Organoid ELISA	2023
10.26275/j48r-vn5s	Patel, B.	Pilot Fecobionics study in healthy human subjects	2023

DOI	Owner	Title	Year
10.26275/ekv2-ohrk	Patel, B.	Fecobionics study in healthy human subjects	2023
10.26275/dm5l-tspb	Armitage, O.	Influence of vagus nerve stimulation on cardiac activity in pigs	2023
10.26275/iv1j-x1g4	Brookes, S.	Characterization of projections of long interneurons in human colon	2023
10.26275/rmcz-jfoq	Nguyen, D.	Anterograde tracing of spinal afferent innervation in flat-mounts of the rat stomach	2023
10.26275/ikfe-z0u7	Makhamreh, A.	Pseudouridine Quantification Data	2023
10.26275/btt6-puqw	Pace, J.	In Vivo Labeling and Detection of Circulating Tumor Cells in Mice Using OTL38	2023
10.26275/d488-z2q8	Verheyden, J.	Identification of lung innervating sensory neurons and their target specificity in mouse (2)	2023
10.26275/5xqz-0dpe	Iavarone, E.	SPARC Metadata Editor Tutorial – Creating new SDS Datasets	2023
10.26275/poex-tria	Iavarone, E.	QuiltedTutorials 2 - Re-Sampling data for computational simulations	2023
10.26275/nuwh-mtsd	Iavarone, E.	SPARClet - Interactive flatmaps	2023
10.26275/f1pu-do9y	Iavarone, E.	SPARC Metadata Editor – Tutorials	2023
10.26275/5yvu-tr0d	Iavarone, E.	Cardiovascular system model for comprehending the acute cardiac effects of vagus nerve stimulation	2023
10.26275/3vqo-xux0	Lin, M.	Scaffold map - Mapping of human gastric enteroendocrine cells	2023

DOI	Owner	Title	Year
10.26275/q8wb-a9qe	Ivich, F.	Ratiometric fluorescence sensing and quantification of circulating blood sodium sensors in mice in vivo	2023
10.26275/8zuc-gst1	Aguinaga, D.	Mid-lumbar (L3) epidural stimulation effects on bladder and external urethral sphincter in non-injured and chronically transected urethane-anesthetized rats	2023
10.26275/gfwu-pi0p	Haberbusch, M.	MicroCT imaging of the fascicular structure in the porcine right and left cervical vagus nerve	2023
10.26275/swkm-bzmg	Gould, T.	Calcium imaging tension recording and pellet transit in mouse colon in response to stimulation of the pelvic nerve (PNS)	2023
10.26275/xnv0-gtsj	Gould, T.	Optogenetic activation of nitrergic and cholinergic neurons of murine colonic myenteric plexus	2023
10.26275/g9qv-rl2d	Iavarone, E.	QuiltedTutorials 1 – Mapping 2D Data to 3D Organ Scaffold	2023
10.26275/efbj-8evl	Blanz, S.	Stimulation of the pig vagus nerve to modulate target effect versus side effect	2023
10.26275/litx-swak	Lin, M.	Scaffold map - Quantification of rat gastric enteroendocrine cells	2023
10.26275/puzi-xtm3	Brookes, S.	Antibodies tested in the colon – Human	2023
10.26275/aqri-vyb4	Brookes, S.	Targets of sympathetic nerves in myenteric plexus of human colon	2023
10.26275/rhda-nblc	Yuan, P.-Q.	CLARITY and 3D imaging with high resolution and deep scanning of innervation in the pig colon by using SP8 DIVE fully tunable spectral multiphoton microscope	2023

DOI	Owner	Title	Year
10.26275/pa9i-likc	Michel, K.	Ussing chamber experiments for distension evoked secretion in human colon	2023
10.26275/3q5o-leo5	Phillips, R.	Effect of chronic gastric electrical stimulation on the feeding behavior of diet-induced obese male Sprague-Dawley rats consuming a 45% high-fat diet	2023
10.26275/ryft-516s	Dinning, P.	High resolution manometry	2023
10.26275/qjpu-t0sy	Lin, M.	Scaffold map - Quantitative analysis of enteric neurons containing choline acetyltransferase and nitric oxide synthase immunoreactivities in the submucosal and myenteric plexuses of the porcine colon	2023
10.26275/n3uf-5doz	Larauche, M.	Influence of acute celiac branch of abdominal vagus nerve stimulation on colonic motility in anesthetized male Yucatan minipigs	2023
10.26275/abac-rzbv	Larauche, M.	Influence of acute thoracolumbar root nerves electrical stimulation on colonic motility in anesthetized male Yucatan minipigs	2023
10.26275/6lqa-ghyr	Tache, Y.	Influence of direct colon tissue electrical stimulation on colonic motility in anesthetized male Yucatan minipig	2023
10.26275/d41u-sokg	Patel, B.	Triaxial mechanical testing of dog colon segments	2023
10.26275/wabs-qul8	Vadigepalli, R.	Closed-loop modeling of central and intrinsic cardiac nervous system circuits underlying cardiovascular control	2023
10.26275/gc2e-qd0w	Pattnaik, A.	Seizure severity score	2023
10.26275/zpxh-rqlw	Iavarone, E.	BIOS Health Vagus Nerve Stimulation Calibrator	2023

DOI	Owner	Title	Year
10.26275/cdjq-pjlb	Iavarone, E.	A multiscale predictive digital twin for neurocardiac modulation in rabbits	2023
10.26275/6b53-usyr	Ardell, J.	RNA sequencing reveals novel transcripts from sympathetic stellate ganglia during cardiac sympathetic hyperactivity in rats	2023
10.26275/fi87-3n6o	Stebbing, M.	Distribution and coexpression patterns of specific cell markers of enteroendocrine cells in pig gastric epithelium	2023
10.26275/59t4-jlnz	Upadhye, A.	Human cervical vagus nerve fascicle imaging with MicroCT	2023
10.26275/dhe4-zh53	Iavarone, E.	Multi-scale human cardiac electrophysiology models	2023
10.26275/tfdf-w3hq	Iavarone, E.	Expression Data Visualization tool	2023
10.26275/s00b-gket	Iavarone, E.	Multi-scale rabbit cardiac electrophysiology models	2023
10.26275/f5se-ynpk	Settell, M.	Histology of pig cervical vagus nerve	2023
10.26275/do5j-mz5q	Rodrigues, A.	Decoding vagus nerve activity with carbon nanotube sensors in freely moving rodents	2023
10.26275/zsop-bygv	Bayrer, J.	Visceromotor responses (VMR) to colorectal distension in mice with silenced or activated enterochromaffin cells	2023
10.26275/wh9h-tbew	Bizanti, A.	Topographical mapping of sympathetic postganglionic innervation of the mouse heart	2023
10.26275/53cg-hai1	Patel, B.	Computational-based mechanical analysis of Fecobionics data	2023
10.26275/yh5c-5pjy	Cheng, Z.	Comparison of the intrinsic cardiac nervous system across male and female rat hearts	2023

DOI	Owner	Title	Year
10.26275/lk0e-kgrs	Pelot, N.	Population of mock morphological models of vagus nerve stimulation with cuff electrodes for the purpose of studying the effect of fascicle diameter on activation threshold	2023
10.26275/ncok-1cof	Pelot, N.	Computational model of heart rate modulation in mice during vagus nerve stimulation	2023
10.26275/kj2u-2ukf	Gould, T.	Chemogenetic activation or inhibition of cholinergic or nitrergic myenteric neurons of mouse colon	2023
10.26275/nomg-p2vk	Ardell, J.	Cardioneural recordings using floating multi-channel plunge micro-electrodes in pigs	2023
10.26275/e3n7-vei4	Pelot, N.	Computational model of laryngeal muscle activation in mice during vagus nerve stimulation	2023
10.26275/sbxk-fwbu	Iavarone, E.	Kember Cardiac Nerve Model	2023
10.26275/srm7-no3j	Damaser, M.	Acute wired Urological Monitor of Conscious Activity (UroMOCA) implantation in feline bladder	2023
10.26275/6f3g-wvzh	Damaser, M.	Acute Wired Colonic Monitor of Conscious Activity (ColoMOCA) implantation in pig bowel	2023
10.26275/z1wa-spub	Hoover, D.	Regional analysis of autonomic nerves in normal and diseased human hearts	2023
10.26275/ggj4-agvt	Smith-Edwards, K.	Myenteric neuron activity during spontaneous motor complexes in mouse colon	2023

DOI	Owner	Title	Year
10.26275/kgkj-6vb9	Feng, B.	Calcium imaging of mouse dorsal root ganglion (DRG) neurons in response to chemical stimuli of distal colon and rectum (colorectum)	2023
10.26275/dfk5-6w3z	Horn, C.	Selective stimulation of the ferret abdominal vagus nerve with multi-contact nerve cuff electrodes	2023
10.26275/d3jb-pkzx	Harvey, R.	Sympathetic and parasympathetic effects on action potentials in isolated pig ventricular myocytes	2023
10.26275/lck3-f9v0	Taylor-Clark, T.	Mapping of the vagal afferent innervation of the mouse lung	2023
10.26275/tpu4-kvzo	Jaffey, D.	Spatial distribution and morphometric characterization of mucosal afferents of the pylorus of the rat stomach	2023
10.26275/x1ht-tsiz	Stebbing, M.	Innervation of enteroendocrine cells in the gastric mucosa in human and pig - including a description of the innervation of mucosal vasculature	2023
10.26275/f4p0-d1ic	Rossen, N.	Mouse genetic models to manipulate enterochromaffin cell activity	2023
10.26275/h4yk-volr	Gould, T.	Calcium imaging and tension recording in response to stimulation of the vagus nerve (VNS)	2023
10.26275/bcrr-uenq	Garg, R.	Defining neuromechanical mechanisms of Achilles tendinopathy progression- healthy human subjects (pilot)	2022
10.26275/vsez-uc77	Ekhlasi, Z.	Generic rat brainstem scaffold	2022
10.26275/xcot-iplx	Christie, R.	Generic pig heart scaffold	2022

DOI	Owner	Title	Year
10.26275/zfpg-gxgg	Soltani, E.	Pig whole-body with embedded organs using automatic workflow for inserting the organs	2022
10.26275/g8aq-rjsp	Brookes, S.	Characterization of projections of longitudinal muscle motor neurons in human colon	2022
10.26275/ueic-1afx	Soltani, E.	Rat whole-body with embedded organs	2022
10.26275/zkol-rciv	Soltani, E.	Rat whole-body with embedded organs using automatic workflow for inserting the organs	2022
10.26275/ewog-btml	Soltani, E.	Mouse whole-body with embedded organs	2022
10.26275/ffnm-sxbj	Soltani, E.	Rat whole-body scaffold	2022
10.26275/5mkx-apz9	Soltani, E.	Human whole-body with embedded organs	2022
10.26275/biqn-mqy4	Smith-Edwards, K.	Enteric neuron responses in mouse distal colon to lumbosacral spinal cord stimulation	2022
10.26275/4iqi-hazf	Ludwig, K.	Anatomy and histology of the domestic pig in the context of vagus nerve stimulation	2022
10.26275/ltks-9kaw	Ekhlasi, Z.	Generic mouse brainstem scaffold	2022
10.26275/hmwa-nqdu	Aristovich, K.	Organotopic organization of the porcine vagus nerve	2022
10.26275/ujsw-77cr	Christie, R.	Generic mouse heart scaffold	2022
10.26275/ajhr-1eom	Ekhlasi, Z.	Generic pig brainstem scaffold	2022
10.26275/0kpx-xh0x	Christie, R.	Generic rat heart scaffold	2022
10.26275/hwgn-8mq4	Ekhlasi, Z.	Generic sheep brainstem scaffold	2022

DOI	Owner	Title	Year
10.26275/pgs1-xfs5	Christie, R.	Generic human heart scaffold	2022
10.26275/ezuv-o6gg	Ekhlasi, Z.	Generic human brainstem scaffold	2022
10.26275/qmg0-zbde	Gould, T.	Calcium imaging tension recordings and pelet transit in mouse colon in response to direct electrical field stimulation	2022
10.26275/eikd-dg5c	Yuan, P.-Q.	RNA sequencing analysis of transcriptomic responses to vagal nerve stimulation in myenteric ganglia of porcine colon	2022
10.26275/2yv6-g6vh	Stacey, W.	Network dynamics of HFOs	2022
10.26275/hbuu-caud	Gaunt, R.	Lower urinary tract nerve responses to high-density epidural spinal cord stimulation in cats	2022
10.26275/05g0-faru	Iavarone, E.	SPARC Metadata Editor (sparc-me) – Tutorials	2022
10.26275/yjtg-qxem	Iavarone, E.	SPARC Metadata Editor (sparc-me) - Creating new SDS datasets	2022
10.26275/pbrd-ci3l	Ardell, J.	Identification of peripheral neural circuits that regulate heart rate using optogenetic and viral vector strategies	2022
10.26275/wcwr-hmch	Damaser, M.	Chronic wireless Urological Monitor of Conscious Activity (UroMOCA) implantation in feline bladder	2022
10.26275/iefx-c2qi	Lin, M.	Generic rat stomach scaffold	2022
10.26275/jnng-wbke	Ekhlasi, Z.	Generic rat lung scaffold	2022
10.26275/zfbf-g88t	Lin, M.	Generic pig stomach scaffold	2022
10.26275/ugeq-kjky	Ekhlasi, Z.	Generic pig lung scaffold	2022
10.26275/in3y-t0ml	Lin, M.	Generic pig colon scaffold	2022

DOI	Owner	Title	Year
10.26275/u8bh-s6be	Lin, M.	Generic mouse stomach scaffold	2022
10.26275/nlfj-rhfk	Ekhlasi, Z.	Generic mouse lung scaffold	2022
10.26275/z1ga-dflp	Lin, M.	Generic mouse colon scaffold	2022
10.26275/aijr-zl44	Ekhlasi, Z.	Generic human lung scaffold	2022
10.26275/uhxm-wxpo	Lin, M.	Generic human esophagus scaffold	2022
10.26275/7ph7-1ez4	Lin, M.	Generic human colon scaffold	2022
10.26275/mxnk-4tvt	Lin, M.	Scaffold map - Influence of direct colon tissue electrical stimulation on colonic motility in anesthetized male Yucatan minipig	2022
10.26275/amsj-jner	Pace, J.	Design and Validation of Near-Infared Diffuse In Vivo Flow Cytometry	2022
10.26275/umgm-rzar	Howard, M.	Correlated electrophysiological immunohistochemical and morphological properties of proximal colon myenteric neurons	2022
10.26275/ebql-cdno	Phillips, R.	Effect of chronic gastric electrical stimulation on the feeding behavior of male rats consuming a 45% high-fat diet	2022
10.26275/spcl-epsl	Liu, Z.	Effects of vagal afferent blockade on gastric motility during cervical vagus nerve stimulation measured with magnetic resonance imaging in rats	2022
10.26275/adzn-2fes	Liu, Z.	In vivo mapping of gastric electrical activation with manganese enhanced magnetic resonance imaging	2022

DOI	Owner	Title	Year
10.26275/r5gw-clgv	Liu, Z.	Effects of vagal efferent blockade on gastric motility and emptying during cervical vagus nerve stimulation measured with magnetic resonance imaging in rats	2022
10.26275/y4k2-mkam	Phillips, R.	Effect of chronic gastric electrical stimulation on the feeding behavior of female rats consuming a 45% high-fat diet	2022
10.26275/z4wa-dxjx	Liu, Z.	Acute effects of gastric electrical stimulation settings on neural activity in the nucleus of solitary tract in rats	2022
10.26275/yt5s-pt6t	Keast, J.	Central terminal fields of lower urinary tract afferents in rat	2022
10.26275/eedx-wrhi	Zeltser, L.	Expression of molecular markers in subpopulations of mouse superior cervical ganglion neurons	2022
10.26275/s3iw-km0k	Zeltser, L.	Expression of molecular markers in subpopulations of mouse celiac ganglion neurons	2022
10.26275/pfjd-cfty	Ardell, J.	Optical mapping of action potentials and calcium transients in the mouse heart during optogenetic stimulation of the intracardiac ganglia and interconnecting neurons (ICNS)	2022
10.26275/bvpu-cuz7	Zeltser, L.	Single cell RNA sequencing of retrogradely labeled mouse stellate ganglion neurons	2022
10.26275/vm1h-k4kq	Ludwig, K.	Electrode design characterization for electrophysiology from swine peripheral nervous system	2022
10.26275/zly9-ow0w	Zeltser, L.	Expression of molecular markers in subpopulations of mouse stellate ganglion neurons	2022

DOI	Owner	Title	Year
10.26275/vkvk-3hqe	Zeltser, L.	Expression of molecular markers in mouse and human stellate ganglia	2022
10.26275/23je-ute3	Jaffey, D.	4D upper gastrointestinal magnetic resonance imaging in healthy human subjects and gastroparetic patients	2022
10.26275/dtlj-t4bf	Tompkins, J.	Increased arrhythmia susceptibility in type 2 diabetic mice related to dysregulation of ventricular sympathetic innervation	2022
10.26275/jrj4-zrmj	Nguyen, D.	Substance P-immunoreactive axon innervation of mouse stomach	2022
10.26275/akxk-vcbm	Rangel, A.	Electrochemical measurement of kanamycin in whole blood for implant longevity evaluation	2022
10.26275/jr6t-jcoe	Gregersen, H.	Performance testing of the Fecobionics device	2022
10.26275/wjll-mekr	Soltani, E.	Human whole-body with embedded organs using automatic workflow for inserting the organs	2022
10.26275/qq9z-e96l	Soltani, E.	Human whole-body scaffold	2022
10.26275/oujt-ao7w	Ekhlasi, Z.	Generic rat bladder scaffold	2022
10.26275/i6uj-vhx8	Ludwig, K.	In vivo visualization of pig vagus nerve 'vagotopy' using ultrasound	2022
10.26275/tscp-vk1d	Gould, T.	Activity of and neurochemical expression in nitrergic and cholinergic neurons in the murine colonic myenteric plexus	2022
10.26275/ya41-6sfo	Feng, B.	Dorsal root ganglion stimulation to modulate mechanosensitive colorectal afferent transmission	2022

DOI	Owner	Title	Year
10.26275/iwxw-y2tz	Liu, Z.	Assessment of gastric emptying and motility with magnetic resonance imaging (MRI) under gastric electrical stimulation (GES) in rats	2022
10.26275/36ua-upkq	Feng, B.	Dorsal root ganglion stimulation to modulate mechanosensitive colorectal afferent transmission in mice	2022
10.26275/x0oc-7oc5	Gould, T.	Calcium imaging and motility tracking of distinct myenteric neuronal subsets in mice	2022
10.26275/luv2-omyk	Balachandran, K.	Generic pig bladder scaffold	2022
10.26275/14qt-e5f0	Ekhlasi, Z.	Generic cat bladder scaffold	2022
10.26275/mwdf-paqm	Balachandran, K.	Generic mouse bladder scaffold	2022
10.26275/qthf-0lou	Ekhlasi, Z.	Generic human bladder scaffold	2022
10.26275/wcli-rv5b	Ardell, J.	Evaluating spheres of influence for efferent neural control of the heart	2022
10.26275/iyto-oxay	Brierley, S.	Imaging colon and bladder sensory convergence in CLARITY cleared mouse spinal cord	2022
10.26275/be0x-9mzy	Brierley, S.	Mapping colon and bladder innervating sensory neurons in CLARITY cleared ganglia in mouse	2022
10.26275/4mfy-y7bj	Aristovich, K.	Temporal dispersion in porcine subdiaphragmatic nerves ex vivo	2022
10.26275/rmkt-5ypu	Hanna, P.	Ablation of the intrinsic cardiac nervous system to evaluate efferent control of cardiac function	2022

DOI	Owner	Title	Year
10.26275/vzxw-kwdu	Bruns, T.	High-density penetrating array dorsal root ganglia recordings	2022
10.26275/x10i-9c9u	Pelot, N.	Human vagus nerve TH- (tyrosine hydroxylase) and ChAT- (choline acetyltransferase) positive fibers	2022
10.26275/nav5-oeol	Pelot, N.	Rat vagus nerve TH- (tyrosine hydroxylase) and ChAT- (choline acetyltransferase) positive fibers	2022
10.26275/zuwb-qnqk	Howard, M.	Calcium dynamics imaging of vasoactive intestinal peptide-expressing (VIP) enteric nervous system (ENS) neurons	2022
10.26275/qw1u-zxea	Nicolai, E.	Vagus nerve stimulation mapping in swine	2022
10.26275/jsaw-2w1q	Harvey, R.	Sympathetic and parasympathetic effects on membrane currents in isolated pig ventricular myocytes	2022
10.26275/1uno-tynt	Wang, L.	Multicolor adeno-associated virus sparse labeling and 3D digital tracing of enteric plexus in mouse proximal colon	2022
10.26275/sip4-ioyz	Yuan, P.-Q.	CLARITY and three-dimensional (3D) imaging of the mouse and porcine colonic innervation	2022
10.26275/rt8g-gu2v	Havton, L.	Ultrastructural analysis of human vagus nerve	2022
10.26275/qskp-awpu	Gould, T.	Optogenetic inhibition of nitrergic and cholinergic neurons of murine colonic myenteric plexus	2022
10.26275/az1n-uv7s	Moss, A.	Spatially tracked single-cell-scale RNAseq of porcine right atrial ganglionic plexus (RAGP) neurons	2022
10.26275/cxej-bm2v	Heuckeroth, R.	Enteric nervous system expression profiling by high throughput scRNA-sequencing in human and mouse colon	2022

DOI	Owner	Title	Year
10.26275/elbl-3vxh	Phillips, R.	Effect of electrical stimulation of vagal afferent terminals located in the stomach muscle wall on feeding behavior	2022
10.26275/wucy-ljuk	Verheyden, J.	Single nucleus RNAseq of nodose ganglia in mice	2022
10.26275/dhbx-w17y	Gregersen, H.	Safety testing of predicate device for Fecobionics	2022
10.26275/of13-iokw	Yuan, P.-Q.	Antibodies tested in the colon - Pig	2022
10.26275/z6jn-j5tx	Moss, A.	Transcriptional diversity of single neurons in the porcine right atrial ganglionic plexus (RAGP)	2022
10.26275/9ly6-az0n	Zhuang, K.	SPARC Nerve Activity Predictor (SNAP)	2022
10.26275/s8jn-wboz	Zhuang, K.	Small Enteric Neural Network Simulator	2022
10.26275/oim6-urls	Zhuang, K.	3D Visualization	2022
10.26275/jetk-pppi	Zhuang, K.	2D Plot	2022
10.26275/frbr-8uyt	Zhuang, K.	opencor-py model	2022
10.26275/gvzt-oeti	Khalifa, F.	Functional mapping with lumbosacral epidural stimulation for restoration of bladder function after spinal cord injury in rats	2022
10.26275/wlhm-c7ba	Stanley, S.	Characterization of adeno associated virus serotypes 4 weeks after pancreas injection in mice	2022
10.26275/lok5-wje6	Morotti, S.	Sympathetic nerve stimulation of mouse and rabbit hearts	2022
10.26275/ih2m-pphy	Liu, Z.	Acute effects of vagus nerve stimulation (VNS) settings on neural activity in the nucleus of solitary tract (NTS) in rats	2022
10.26275/by2v-qren	Zhuang, K.	Jupyterlab exploration of computational models of unmyelinated peripheral axons	2022

DOI	Owner	Title	Year
10.26275/9uqz-zwnh	Liu, Z.	Acute effects of efferent and afferent vagus nerve stimulation (VNS) on neural activity accessed with functional Magnetic Resonance Imaging (fMRI) in rats	2022
10.26275/tbuz-s6gu	Liu, Z.	Acute effects of gastric electrical stimulation settings on gastric motility assessed with magnetic resonance imaging in rats	2022
10.26275/g3xc-oztw	Sternini, C.	Quantitative analysis of enteric neurons containing choline acetyltransferase and nitric oxide synthase immunoreactivities in the submucosal and myenteric plexuses of the porcine colon	2022
10.26275/chfk-eugm	Keast, J.	ViNERS (Visceral Nerve Ensemble Recording & Stimulation) peripheral neural interface modeling environment	2021
10.26275/s2vo-pje2	Ardell, J.	Identification of peripheral neural circuits that regulate heart rate using optogenetic and viral vector strategies part (2)	2021
10.26275/kkmb-vun5	Keast, J.	Recording of electrically evoked neural activity and bladder pressure responses in awake rats chronically implanted with a pelvic nerve array	2021
10.26275/ts6z-z80x	Gregersen, H.	Safety testing of the Fecobionics device	2021
10.26275/e6vk-2lky	Verheyden, J.	Identification of lung innervating sensory neurons and their target specificity in mouse (1)	2021
10.26275/z61u-2tcs	Keast, J.	Simulations of pelvic and vagus neural interface anatomy-dependent stimulus and recording properties	2021
10.26275/ypwk-0xbo	Liu, Z.	Acute effects of gastric electrical stimulation (GES) settings on neural activity accessed with functional magnetic resonance imaging (fMRI) in rats	2021

DOI	Owner	Title	Year
10.26275/ryap-anvh	Ward, M.	An interactive system to visualize propagating vagal nerve activity in response to gastric electrical stimulation for gastroparesis	2021
10.26275/eoqv-ozxc	Liu, Z.	Acute effects of efferent and afferent vagus nerve stimulation (VNS) on neural activity accessed with functional Magnetic Resonance Imaging (fMRI) in rats (Part 2)	2021
10.26275/fsfw-dmfd	Chen, P.	Effects of subcutaneous nerve stimulation with blindly inserted electrodes on ventricular rate control in a canine model of persistent atrial fibrillation	2021
10.26275/c14g-2czn	Moss, A.	Spatially tracked single-neuron transcriptomics of a male porcine right atrial ganglionic plexus (RAGP)	2021
10.26275/slsc-eahw	Moss, A.	Spatially tracked single-neuron transcriptomics of a female porcine right atrial ganglionic plexus (RAGP)	2021
10.26275/hrww-enzr	Moss, A.	Spatially tracked single-cell transcriptomics map of neuronal networks in the intrinsic cardiac nervous system	2021
10.26275/lzhy-ox20	Bernabei, J.	HUP Interictal iEEG Atlas	2021
10.26275/9ffg-482d	Howard, M.	3D imaging of enteric neurons in mouse	2021
10.26275/j5wc-rwcr	Bruns, T.	Intraneural recordings in rat vagus nerves using carbon fiber microelectrode arrays	2021
10.26275/mhq6-csy1	Muenzberg, H.	iWAT (inguinal white adipose tissue) sympathetic innervation circuit pseudorabies viral tracing in reporter mice	2021
10.26275/drlw-j2cp	Zhuang, K.	Jupyter Voila Interactive Data Analysis	2021

DOI	Owner	Title	Year
10.26275/t8he-z5uu	Liu, Z.	Effects of nodose ganglion blockade on gastric motility during cervical vagus nerve stimulation measured with magnetic resonance imaging in rats	2021
10.26275/do5j-enxl	Muenzberg, H.	Monosynaptic circuit mapping of iBAT (interscapular brown adipose tissues) in mice	2021
10.26275/pb3l-251h	Leung, C.	Mapping of intrinsic cardiac nervous system (ICN) neurons in a 3D reconstructed rat heart	2021
10.26275/pvib-4jat	Muenzberg, H.	Endorgan-specific Pseudorabies (PRV) infection in mouse kidney and liver	2021
10.26275/ofja-ghoz	Pelot, N.	Quantified morphology of the human vagus nerve with anti-claudin-1	2021
10.26275/er7m-gir3	Khalifa, F.	Functional mapping with lumbosacral epidural stimulation for restoration of bladder function after spinal cord injury in rats (T13)	2021
10.26275/qcuk-a8ty	Ludwig, K.	Sources of off-target effects for vagus nerve stimulation using the LivaNova clinical lead in swine	2021
10.26275/ngey-3iz7	Chen, P.	Effects of subcutaneous nerve stimulation on nerve sprouting in ambulatory dogs	2021
10.26275/wyn1-eww6	Howard, M.	3D imaging of enteric neurons in a male mouse	2021
10.26275/lkvz-vrcy	Yuan, P.-Q.	Single cell RNA sequencing (scRNAseq) analysis identifies the cell populations in the muscularis externa of the pig colon	2021

DOI	Owner	Title	Year
10.26275/ek1m-xqw1	Harvey, R.	Sympathetic and parasympathetic effects on subcellular cAMP responses in isolated ventricular myocytes	2021
10.26275/i7dl-58h1	Wang, L.	Antibodies tested in the colon – Mouse	2021
10.26275/j4he-9spq	Muenzberg, H.	Optogenetic iBAT (interscapular brown adipose tissue) stimulation in anesthetized mice	2021
10.26275/1h3s-thms	Muenzberg, H.	Sympathetic iBAT (interscapular brown adipose tissue) activation high fat (HF) low fat (LF) diet study	2021
10.26275/jkux-orfg	Fuller, D.	Phrenic nerve stimulation spinal intact rats	2021
10.26275/0khe-2os4	Mazzuoli-Weber, G.	In vitro imaging of mechanosensitive submucous neurons in the porcine colon	2021
10.26275/imbg-0okx	Damaser, M.	Effects of cystotomy on the feline urinary bladder	2021
10.26275/advv-1awo	Sladek, J.	Optogenetic stimulation prevents lipopolysaccharide induced TNFa production	2021
10.26275/ckgb-5ewo	Muenzberg, H.	TRAP-SEQ (Translating Ribosome Affinity Purification followed by RNA sequencing) of interscapular brown adipose tissue (iBAT)- related ganglia from 7-day cold and warm treated mice	2021
10.26275/8vi1-tcsp	Morris, K.	Tool for uploading files to Blackfynn	2021
10.26275/56h4-ypua	Moss, A.	Acquisition of single neurons and regional neuronal samples from the porcine right atrial ganglionic plexus (RAGP) through laser capture microdissection	2021

DOI	Owner	Title	Year
10.26275/pkgd-bopz	Muenzberg, H.	Retrograde tracing of interscapular brown adipose tissue (iBAT) specific sympathetic neurons in mice - virus and reporter testing	2021
10.26275/m9ti-0pbj	Muenzberg, H.	Gene expression profile of interscapular brown adipose tissue (iBAT) and inguinal white adipose tissue (iWAT) whole ganglia sequencing in mice	2021
10.26275/pidf-15l3	Muenzberg, H.	Chemogenetic whole-body and iBAT (interscapular brown adipose tissue) -specific sympathetic stimulation in anesthetized mice	2021
10.26275/tuof-9odl	Muenzberg, H.	Chemogenetic iBAT (interscapular brown adipose tissue)-specific sympathetic stimulation and e-mitter implant in mice	2021
10.26275/rtzw-x9u4	Powley, T.	Functional mapping of the stomach neural circuitry - gastric electrical stimulation (GES) evoked duodenal motility in rats	2021
10.26275/zxe9-o3ss	Powley, T.	MicroCT imaging of rat stomach vasculature with Microfil MV-122	2021
10.26275/6xtv-zfpc	Scheller, E.	Spatial mapping and contextualization of axon subtypes innervating the long bones of C3H and B6 mice	2021
10.26275/w027-cisv	Yang, H.	Imaging in vivo acetylcholine release in the peripheral nervous system with a fluorescent nanosensor in mice	2021
10.26275/jl5t-xfgu	Powley, T.	Micro Computed Tomography (Micro-CT) imaging of iodine-stained rat stomachs from full to empty	2021
10.26275/gdot-t59p	Keast, J.	Immunohistochemical classification of sensory and autonomic neurons projecting to the lower urinary tract in rats	2021

DOI	Owner	Title	Year
10.26275/fcrd-lbid	Campbell-Thompson, M.	Human islet microvasculature analysis	2021
10.26275/dap3-ckep	Pelot, N.	Pig vagus nerve TH (tyrosine hydroxylase) and ChAT (choline acetyltransferase) positive fibers	2021
10.26275/bjp1-ppqo	Vaseghi, M.	Functional neuronal nodose recording from pig - Cardiac field chemical and mechanical stimulation	2020
10.26275/mguq-j2n3	Nanivadekar, A.	Selectivity of afferent microstimulation at the DRG using epineural and penetrating electrode arrays	2020
10.26275/x9xq-e4wu	Williams, A.	LLC and MM DiFC CTC detections 2020_11_04	2020
10.26275/tv7g-o8ff	Clancy, C.	A multi-scale model of cardiac electrophysiology	2020
10.26275/ilkm-9f8r	Biscola, N.	Morphometric analysis of the abdominal vagus nerve in rats	2020
10.26275/maq2-eii4	Pelot, N.	Quantified morphology of the pig vagus nerve	2020
10.26275/sydt-lkiw	Pelot, N.	Human vagus nerve stained with Masson's trichrome	2020
10.26275/ilb9-0e2a	Pelot, N.	Quantified morphology of the rat vagus nerve	2020
10.26275/8pc2-rhu2	Pelot, N.	Quantified morphology of the pig vagus nerve with anti-fibronectin	2020
10.26275/jg3k-z5qm	Keast, J.	Immediate early gene (IEG) mapping of spinal cord neurons activated by cystometry induced micturition in rat	2020
10.26275/0y4e-eskx	Gould, T.	Distribution of nitregic cholinergic and all myenteric plexus neurons	2020
10.26275/k64z-n56w	Wagenaar, J.	Scientific Articles	2020

DOI	Owner	Title	Year
10.26275/kw6v-vftb	Pascual, I.	Neurofauna Rat	2020
10.26275/boe7-1bms	Horn, C.	Characterizing the effect of feeding distension and emetic stimuli on gastric myoelectric activity in ferrets	2020
10.26275/z3ab-7j9y	Pelot, N.	Rat vagus nerve stained with Masson's trichrome	2020
10.26275/pgr9-bk2e	Pelot, N.	Pig vagus nerve stained with Masson's trichrome	2020
10.26275/hgwy-pchm	Calderon, I.	Real-time particle-by-particle detection of erythrocyte camouflaged microsensor with extended circulation time in the bloodstream	2020
10.26275/owri-mpsx	Ardell, J.	Functional recordings from the pig intrinsic cardiac nervous system (ICN)	2020
10.26275/nnyt-bqpg	Mitchell, G.	Phrenic nerve immunohistochemistry	2020
10.26275/prjd-jhoc	Clark, H.	Submandibular ganglion stained by bungarotoxin and nanosensors in mouse	2020
10.26275/63lh-hdz5	Chen, P.	Electrophysiology in dog after subcutaneous nerve stimulation	2020
10.26275/xkoa-oqec	Muenzberg, H.	iBAT (interscapular Brown Adipose Tissue) sympathetic innervation circuit pseudorabies viral tracing in reporter mice	2020
10.26275/4qvr-kwzq	Schemann, M.	Ussing chamber pressure pump	2020
10.26275/osy6-dn3o	Helmer, K.	Bilateral recordings of cervical vagus nerve activity in rats	2020
10.26275/t6j6-77pu	Wagenaar, J.	Hetionet	2020
10.26275/uztw-z5sc	Clancy, C.	Prototype simulation of undiseased human cardiac ventricular cells	2020

DOI	Owner	Title	Year
10.26275/1upo-xvkt	Morris, K.	Feline brainstem neuron extracellular potential recordings	2020
10.26275/ukz3-0fao	Powley, T.	Spatial distribution and morphometric characterization of vagal efferents associated with the myenteric plexus of the rat stomach	2020
10.26275/3m8n-0owa	Powley, T.	Spatial distribution and morphometric characterization of vagal afferents (intramuscular arrays (IMAs)) within the longitudinal and circular muscle layers of the rat stomach	2020
10.26275/wzry-sf7v	Powley, T.	Spatial distribution and morphometric characterization of vagal afferents associated with the myenteric plexus of the rat stomach	2020
10.26275/spfh-lx9g	Aristovich, K.	Imaging fast neural traffic at fascicular level with electrical impedance tomography - Proof of principle in rat sciatic nerve	2020
10.26275/o9qr-l4x9	Stebbing, M.	Quantification of rat gastric enteroendocrine cells	2020
10.26275/mzth-oxbk	Stebbing, M.	Quantification of the relationship between rat gastric nerve fibers and enteroendocrine cells (EEC)	2020
10.26275/ppgj-qqpf	Stebbing, M.	Mapping of human gastric enteroendocrine cells	2020
10.26275/w4my-puqm	Ardell, J.	Functional neuronal nodose recording from pig- Modulation by myocardial ischemia and variably coupled PVC's	2020
10.26275/xmsp-wwtu	Stanley, S.	Quantification of Cholera Toxin Subunit Beta (CTb) positive neurons in the coeliac nodose and dorsal root ganglia 1 week after pancreas injection in mice	2020

DOI	Owner	Title	Year
10.26275/dwzu-xtmj	Zeltser, L.	Cholera toxin B retrograde tracing from brown adipose tissue and forelimb to the stellate ganglion	2020
10.26275/ge74-ypxd	Zeltser, L.	Visualizing sympathetic projections in the intact brown adipose tissue depot in the mouse	2020
10.26275/pzek-91wx	Heuckeroth, R.	Robust 3-Dimensional visualization of human colon enteric nervous system without tissue sectioning	2020
10.26275/c4xq-9kl0	Gonzalez-Rothi, E.	Effect of intermittent hypoxia preconditioning in rats with chronic cervical spinal cord injury – An electrophysiological study	2020
10.26275/mvwc-fnqm	Powley, T.	Vagus nerve stimulation promotes gastric emptying by increasing pyloric opening measured with magnetic resonance imaging	2020
10.26275/guqw-r3ca	Helmer, K.	Processed fMRI data of transcutaneous auricular vagus nerve (taVNS) stimulation in humans	2020
10.26275/qh3q-elj6	Ward, M.	Influence of left vagal stimulus pulse parameters on vagal and gastric activity in rat	2020
10.26275/t4ng-2zm6	McCallum, G.	Chronic interfacing with the autonomic nervous system using carbon nanotube (CNT) yarn electrodes	2020
10.26275/duz8-mq3n	Patel, B.	Computational modelling of the mechanical behavior of the colon	2020
10.26275/mq7u-tdjd	Pascual, I.	Anatomical Model Viewer	2019
10.26275/pfxu-irqf	Johnson, J.	Intracranial EEG Epilepsy - Study 6	2019
10.26275/psj7-wggf	Johnson, J.	Intracranial EEG Epilepsy - Study 3	2019

DOI	Owner	Title	Year
10.26275/hqyv-oagj	Johnson, J.	Intracranial EEG Epilepsy - Study 5	2019
10.26275/ju7v-hnyy	Boccanfuso, J.	Canine Epilepsy Dataset	2019

Review of Neuroscience Platforms

Table S2: Brain-CODE review.

**Data Management Opportunity**	**Comments**
Multimodal Data Management	Supports numerous data types including clinical, neuroimaging, molecular, and multiomics data from humans, animal models, and basic science research.
Comprehensive Metadata Support	Includes metadata on study protocols, equipment, and participant-related information. These inform data access controls and allow data to be combined across research programs.
FAIR Data Sharing	The development of Brain-CODE is directly guided by the FAIR principles. It features data sharing policies, standardization efforts, and a federated data architecture.
Optimizing Data Reliability and Utilization	Multiple data quality assurance and control processes are implemented. It provides tools for data capture and exploration, as well as workspaces with dedicated computing resources for processing and analytics.
Facilitating Data Integration and Standardization	Implements CDEs, ontologies, and standardized processes across studies. It also applies the Clinical Data Interchange Standards Consortium (CDISC) standards where possible.
Fostering Collaborative Science	Data federation and linkages with external databases support data sharing with collaborators. Workspace environments centralize data workflows.
Enabling Scalable Analysis	Provides scalable computing resources and integrated tools for data analysis.
Ensuring Resource Sustainability	Federation with national and international databases, a detailed governance structure, and an extensibly-designed platform help it's long-term utility. The platform is closed-source and hosted at a high-performance computing facility (Centre for Advanced Computing, Ontario, Canada).

Table S3: brainlife.io review.

**Data Management Opportunity**	**Comments**
Multimodal Data Management	Handles multiple neuroimaging modalities including MRI, MEG, and EEG data. While limited in modalities, it improves the interoperability of these data formats from different sources.
Comprehensive Metadata Support	Although brainlife.io tracks data provenance and uses standardized data formats, there is not an explicit focus on comprehensive metadata management.
FAIR Data Sharing	Developed specifically in support of the FAIR principles.
Optimizing Data Reliability and Utilization	Focuses heavily on enabling reliable data processing, analysis, and reuse across multiple neuroimaging modalities through its analysis workflows and tools.
Facilitating Data Integration and Standardization	Implements 'Datatypes' as a key standardization feature that make data interoperable across applications.
Fostering Collaborative Science	Supports both public and private projects. Enables the sharing of apps and computational resources for collaborative research.
Enabling Scalable Analysis	Integrates high-performance computing and cloud resources to support scalable analysis.
Ensuring Resource Sustainability	Operates on a public-funding model and supports a large user base across numerous institutions. The platform is fully open-source and the cloud architecture is described in-detail.

Table S4: DABI review.

Data Management Opportunity	Comments
Multimodal Data Management	Supports various data types including electrophysiology, clinical, imaging, pathology, demographics, and behavioral data.
Comprehensive Metadata Support	Implements metadata management through data harmonization and CDEs. Metadata can be querying and visualized with the ability to create cohorts.
FAIR Data Sharing	While not adhering to the FAIR principles explicitly, DABI implements practices that align them: accessible data sharing, standardized formats for data interoperability, and features that support data reuse.
Optimizing Data Reliability and Utilization	Data provenance is captured and retained alongside the data. Additionally, an audit trail tracks changes made to datasets. Provides tools for data analysis, visualization, and quality control.
Facilitating Data Integration and Standardization	Encourages the use of standardized formats like BIDS and NWB. Metadata files are required, and a metadata harmonization tool is provided for data in non-compliant formats.
Fostering Collaborative Science	Collaborative features on the platform are limited to data sharing and access control.
Enabling Scalable Analysis	Integrates with analytical tools, offers a machine learning ecosystem, and allows for data exploration without needing to download raw data.
Ensuring Resource Sustainability	LONI aims to maintain the archive after funding ends without charging users fees for data access and has historically done so for other projects. The platform is built with a cloud architecture, although it is closed-source.

Table S5: DANDI review.

Data Management Opportunity	Comments
Multimodal Data Management	Accepts electrophysiology, optophysiology, behavioral time-series, and images from immunostaining experiments, among others.
Comprehensive Metadata Support	Supports metadata, stores them together with data, and has a standard schema. Capabilities beyond organization are limited.
FAIR Data Sharing	DANDI is compliant with the FAIR principles and prioritizes open access data. Assigns DOIs to dataset versions for citability.
Optimizing Data Reliability and Utilization	Uploaded data are validated for adherence to standardized formats and it has data identifiers for versioning datasets and assets. Allows programmatic access to data in the cloud and provides built-in tools for organization, visualization, and analysis.
Facilitating Data Integration and Standardization	Encourages the use of data standards (NWB, BIDS, NIDM, and other BRAIN Initiative standards) for consistency and interoperability across datasets.
Fostering Collaborative Science	Projects on DANDI allow collaborators and the integration with JupyterHub potentially enables shared data analyses. Built-in, collaborative features on the platform itself are minimal.
Enabling Scalable Analysis	Allows data streaming for access to parts of datasets. Has compute-near-data functionality through DANDI Hub, enabling users to perform analyses on large datasets without local downloads.
Ensuring Resource Sustainability	A partnership with the BRAIN Initiative and funding from NIH support long-term sustainability. The platform itself is cloud-based and open-source.

Table S6: EBRAINS review.

**Data Management Opportunity**	**Comments**
Multimodal Data Management	Hosts data from all modalities and species, as well as models, software, and atlases.
Comprehensive Metadata Support	A dedicated curation team supports researchers in describing and annotating their data. The platform uses the openMINDS metadata framework for standardized metadata management.
FAIR Data Sharing	EBRAINS is committed to the FAIR principles — providing a platform with open access policies and capabilities for data integration and sharing.
Optimizing Data Reliability and Utilization	Provides long-term storage solutions and emphasizes data reusability. Includes tools for modeling and simulation.
Facilitating Data Integration and Standardization	Data is standardized through the curation process using community-driven metadata standards and ontologies. Consistent and precise organization is prioritized over strict standardization.
Fostering Collaborative Science	Offers collaborative, cloud workspaces for private and community collaboration. Provides tools for sharing and accessing data, software, models, and atlases.
Enabling Scalable Analysis	Provides access to high-performance computing resources for large-scale data analysis and simulations.
Ensuring Resource Sustainability	Provides free data storage at the Swiss National Supercomputing Centre for at least 10 years. Substantial funding from the EU supports long-term sustainability and it is part of the European Open Science Cloud association (EOSC). EBRAINS has open-source elements, but the platform is not fully open-source.

Table S7: The IDA review.

Data Management Opportunity	Comments
Multimodal Data Management	Manages various types of neuroscience data, including MRI, PET, SPECT, and EEG. Also supports multiomics, clinical, and biospecimen data, among other modalities.
Comprehensive Metadata Support	While demographic and clinical metadata are included and searchable, there's not a comprehensive approach to metadata management.
FAIR Data Sharing	The IDA implements practices that align with the FAIR principles including data archiving, exploring, and sharing. However, some FAIR strategies like dataset DOI assignment are not implemented.
Optimizing Data Reliability and Utilization	It ensures reliability and uptime through fault-tolerant network infrastructure. Features advanced search capabilities to build data collections and easy download options.
Facilitating Data Integration and Standardization	Offers tools for de-identifying data and ensures compliance with patient-privacy regulations. It also provides standardized processes for data upload and download. However, there's no broader data standardization efforts across studies.
Fostering Collaborative Science	Designed to facilitate data transfers between collaborative groups. Has an API and tools for external developers to access and integrate data on the IDA. However, collaborative features within the platform are lacking.
Enabling Scalable Analysis	The IDA doesn't directly provide analysis tools, but the API allows external tools and workflows to access data.
Ensuring Resource Sustainability	Widely used for the long-term preservation of neuroscience and biomedical research data. Although the platform is closed-source, its infrastructure is robust, and it is supported by major institutions.

Table S8: OpenNeuro review.

Data Management Opportunity	Comments
Multimodal Data Management	Supports MRI, EEG, iEEG, MEG, and PET data, among other BIDS-compatible data types.
Comprehensive Metadata Support	Includes basic metadata through the BIDS format and additional dataset-level metadata can be provided by dataset contributors. While the extent of metadata features is limited by the BIDS specification, this ensures consistency across datasets.
FAIR Data Sharing	Explicitly addresses each FAIR principle through persistent, unique identifiers, multiple data retrieval methods, BIDS standardization, and open data sharing.
Optimizing Data Reliability and Utilization	Offers easy downloading options via web and programming interfaces. Data is well-organized and structured. Uses content-addressing, checksums, and incremental backups to ensure data integrity.
Facilitating Data Integration and Standardization	Strictly adheres to BIDS format, ensuring a high level of standardization across datasets.
Fostering Collaborative Science	Primarily facilitates collaboration through public data sharing. Direct collaborative features within data archives are not prioritized.
Enabling Scalable Analysis	While OpenNeuro itself focuses on data sharing rather than analysis, cloud-based analysis and visualization tools are provided on partner platforms.
Ensuring Resource Sustainability	Receives support and funding through the BRAIN Initiative and other large organizations. The platform is cloud-based and fully open-source.

References

[1] Gardner, A. B. *et al.* Canine epilepsy dataset (version 1) [dataset] (2019). URL `https://doi.org/10.26275/JU7V-HNYY`.

[2] Napadow, V. & Sclocco, R. Processed fmri data of transcutaneous auricular vagus nerve (tavns) stimulation in humans (version 1) [dataset] (2020). URL `https://doi.org/10.26275/GUQW-R3CA`.

[3] Muenzberg, H. *et al.* Trap-seq (translating ribosome affinity purification followed by rna sequencing) of interscapular brown adipose tissue (ibat)-related ganglia from 7-day cold and warm treated mice (version 1) [dataset] (2021). URL `https://doi.org/10.26275/CKGB-5EWO`.

[4] Havton, L. A. *et al.* Morphometric analysis of the abdominal vagus nerve in rats (version 1) [dataset] (2020). URL `https://doi.org/10.26275/ILKM-9F8R`.

[5] Pelot, N. A., Grill, W. M. & Huffman, W. Computational model of heart rate modulation in mice during vagus nerve stimulation (version 1) [dataset] (2023). URL `https://doi.org/10.26275/NCOK-1COF`.

[6] Sukasem, A., Ekhlasi, Z., Hunter, P. & Christie, R. Generic rat lung scaffold (version 3) [dataset] (2022). URL `https://doi.org/10.26275/JNNG-WBKE`.
